# Multiple oxidative post-translational modifications of human glutamine synthetase mediate peroxynitrite-dependent enzyme inactivation and aggregation

**DOI:** 10.1016/j.jbc.2023.102941

**Published:** 2023-01-23

**Authors:** Nicolás Campolo, Mauricio Mastrogiovanni, Michele Mariotti, Federico M. Issoglio, Darío Estrin, Per Hägglund, Tilman Grune, Michael J. Davies, Silvina Bartesaghi, Rafael Radi

**Affiliations:** 1 Departamento de Bioquímica and Centro de Investigaciones Biomédicas (CEINBIO), Facultad de Medicina, Universidad de la República, Montevideo, Uruguay; 2 Department of Biomedical Sciences, Panum Institute, University of Copenhagen, Copenhagen, Denmark; 3 CONICET-Universidad de Buenos Aires, Instituto de Química Biológica de la Facultad de Ciencias Exactas y Naturales (IQUIBICEN), Buenos Aires, Argentina; 4 Instituto de Tecnologia Química e Biológica António Xavier, Universidade Nova de Lisboa (ITQB NOVA), Oeiras, Portugal; 5 CONICET-Universidad de Buenos Aires, Instituto de Química Física de los Materiales, Medio Ambiente y Energía (INQUIMAE), Buenos Aires, Argentina; 6 Departamento de Química Inorgánica, Facultad de Ciencias Exactas y Naturales, Universidad de Buenos Aires, Analítica y Química Física, Buenos Aires, Argentina; 7 Department of Molecular Toxicology, German Institute of Human Nutrition, Potsdam-Rehbrücke, Nuthetal, Germany; 8 German Center for Cardiovascular Research (DZHK), Berlin, Germany; 9 Department of Physiological Chemistry, Faculty of Chemistry, University of Vienna, Vienna, Austria

**Keywords:** glutamine synthetase, oxidants, free radicals, peroxynitrite, hydrogen peroxide, nitrotyrosine, dityrosine, thiol oxidation, aggregation, ABAP, 2,2′-azobis (2-amidinopropane) dihydrochloride, DiTyr, 3,3’-dityrosine, DF, desferrioxamine, GlnNHOH, γ-glutamyl hydroxamate, GS, glutamine synthetase, MRM, Multiple Reaction Monitoring, ONOO^-^, peroxynitrite, UHPLC, ultra high performance liquid chromatography

## Abstract

Glutamine synthetase (GS), which catalyzes the ATP-dependent synthesis of L-glutamine from L-glutamate and ammonia, is a ubiquitous and conserved enzyme that plays a pivotal role in nitrogen metabolism across all life domains. In vertebrates, GS is highly expressed in astrocytes, where its activity sustains the glutamate-glutamine cycle at glutamatergic synapses and is thus essential for maintaining brain homeostasis. In fact, decreased GS levels or activity have been associated with neurodegenerative diseases, with these alterations attributed to oxidative post-translational modifications of the protein, in particular tyrosine nitration. In this study, we expressed and purified human GS (HsGS) and performed an in-depth analysis of its oxidative inactivation by peroxynitrite (ONOO^−^) *in vitro*. We found that ONOO^−^ exposure led to a dose-dependent loss of HsGS activity, the oxidation of cysteine, methionine, and tyrosine residues and also the nitration of tryptophan and tyrosine residues. Peptide mapping by LC-MS/MS through combined H_2_^16^O/H_2_^18^O trypsin digestion identified up to 10 tyrosine nitration sites and five types of dityrosine cross-links; these modifications were further scrutinized by structural analysis. Tyrosine residues 171, 185, 269, 283, and 336 were the main nitration targets; however, tyrosine-to-phenylalanine HsGS mutants revealed that their sole nitration was not responsible for enzyme inactivation. In addition, we observed that ONOO^−^ induced HsGS aggregation and activity loss. Thiol oxidation was a key modification to elicit aggregation, as it was also induced by hydrogen peroxide treatment. Taken together, our results indicate that multiple oxidative events at various sites are responsible for the inactivation and aggregation of human GS.

Glutamine synthetase (GS; EC 6.3.1.2) is a key enzyme in nitrogen-metabolism that catalyzes the ATP-dependent synthesis of L-glutamine from L-glutamate and ammonia (Equation 1). This results in the incorporation of inorganic nitrogen into an organic intermediate that acts as a nitrogen source in multiple metabolic processes, including amino acid and nucleotide biosynthesis. Consequentially, GS enzymes are ubiquitous and conserved across all life domains, from unicellular organisms to higher vertebrates, and is considered an ancestral enzyme (1, 2, 3).

(1) $$L-\text{glutamate}+{\text{NH}}_{3}+\text{ATP}\;\to \;L-\text{glutamine}+\text{ADP}+\text{Pi}$$

Three GS isoforms have been described, based on sequence, structure, and function: GSI, the main prokaryote form; GSII, found mainly in eukaryotes; and GSIII, which is limited to a few prokaryotes. Some prokaryotes also contain GSII, and GSI has been reported in some eukaryotes, consistent with gene duplication before prokaryotes and eukaryotes diverged (3, 4, 5, 6, 7).

The 3-D structure of each type has been determined (1, 8, 9, 10), with each being large homo-oligomers composed of two closed ring structures associated across an interface and arranged with dihedral symmetry (1). The active sites, which are located at the interface between monomers in each ring (9), have a bifunnel shape and contain three divalent cation-binding sites occupied by manganese or magnesium ions (11). GSI and GSIII are dodecamers with two hexamer rings held together by hydrophobic interactions and hydrogen bonds (11). GSII is decamer with two pentamer rings: human GS (HsGS) therefore comprises 10 × 42 kDa monomers, each of 373 amino acids (8, 9).

Most vertebrates express a single GS isoform in a tissue-specific pattern with either cytosolic or mitochondrial localization (12). The GS-catalyzed reaction plays an important role in ammonia detoxification, with perivenous hepatocytes, expressing high GS levels in mitochondria that metabolize ammonia that escapes ureagenesis by periportal hepatocytes. In the central nervous system, GS is highly expressed in the cytosol of astrocytes where it maintains the glutamate-glutamine cycle, with the conversion of glutamate into glutamine at glutamatergic synapses, essential for recycling of this neurotransmitter and avoidance of excitotoxicity (9, 13, 14). As a consequence, changes in the activity or level of GS can result in astroglial dysfunction and adversely affect neuronal survival (15).

Decreased GS levels or activity have been associated with neurodegenerative disorders, including Alzheimer’s disease in humans and in animal models (16, 17, 18, 19, 20). GS activity has been reported to decrease with aging (21, 22), and this has been linked to an increase in the extent of oxidation (*e.g.*, protein carbonylation) of the protein in Alzheimer’s disease, suggesting an association between these phenomena (23, 24, 25, 26, 27). Thus, impairment of the glutamate-glutamine cycle in experimental animals can cause behavioral and mood alterations, disturbance of sleeping patterns, amnesia, confusion, and reduced awareness, that resemble those observed in people with Alzheimer’s disease (16, 28, 29). Neurological alterations can also arise due to a loss of hepatic GS activity as a result of impaired systemic ammonia metabolism (14).

Literature data indicate that GS activity can be decreased by endogenous oxidants (30, 31, 32, 33, 34, 35), supporting the hypothesis that post-translational oxidative modifications may be involved in the loss of activity observed in these pathologies. GS inactivation has been shown to occur *in vitro via* the actions of peroxynitrite (ONOO^−^)†, the product of the diffusion-controlled reaction between nitric oxide (^•^NO) and superoxide anion radical (O_2_^•−^) (36, 37). Thus, Berlett *et al.* reported that *Escherichia coli* GS was inactivated by ONOO^−^, with this attributed to conversion of GS tyrosine (Tyr) residues to 3-nitrotyrosine (NO_2_Tyr) (30, 31). The plant GS1a isoform is also inactivated by ONOO^−^, with this reported to occur *via* nitration of Tyr167 (as indicated by site-directed mutagenesis), though the GS2a isoform appears to be inactivated *via* thiol modification (32). In the case of ovine GS (33, 34), inactivation has been associated with nitration of Tyr336 as determined by peptide-mapping using mass spectrometry. This residue participates in the ATP/ADP-binding during catalysis, consistent with modification impacting on protein function. Molecular dynamics simulations support this hypothesis (38). In both cell systems exposed to inflammatory conditions and some animal models of disease (amyotrophic lateral sclerosis, brain hypoxia-reoxygenation), endogenous GS was found nitrated and, in cell cultures, also with a decreased activity (34, 35, 39, 40, 41).

Although much of the current evidence points to Tyr nitration being (at least partly) responsible for GS inactivation, this may be in part due to the ready availability of assays for NO_2_Tyr and limited methods to assess other mechanisms. Thus, ONOO^−^ and its protonated form, peroxynitrous acid (ONOOH, p*Ka* 6.8), can oxidize cysteine (Cys), methionine (Met), and tryptophan (Trp) residues directly and can also give rise to strong one-electron oxidants, including the carbonate anion radical (CO_3_^•−^), hydroxyl radical (^•^OH), and nitrogen dioxide (^•^NO_2_) *via* homolytic reactions. These radicals can modify many amino acids, including Cys, Met, Trp, Tyr, and histidine (His) (42, 43, 44, 45).

Multiple reports indicate that GS inactivation can occur *via* oxidants other than ONOO^−^ and *via* alternative mechanisms (46, 47, 48, 49). Furthermore, it is unlikely that only single residues (such as Tyr336) would be *selectively* nitrated when pure GS is exposed to ONOO^−^, given the presence of 15 Tyr residues per monomer (43, 50, 51). In addition, there is limited semiquantitative or quantitative data with respect to the extent of Tyr336 modification (and also for other residues), which is critical to addressing whether a biologically relevant loss of activity occurs *via* nitration at this site, especially as Tyr nitration is typically a low yield reaction (37, 50, 52). Finally, the possibility that GS oxidation by ONOO^−^ (and other oxidants) may affect protein function by mechanisms other than impaired catalysis (*e.g.*, *via* protein unfolding and/or aggregation) has not been explored.

In view of these knowledge gaps, we have performed an in-depth analysis of the oxidative inactivation of pure recombinant HsGS by ONOO^−^, using a combination of biochemical, analytical, molecular biology, structural, and proteomic methodologies. The results suggest much more complex mechanisms involved in its loss of function than envisaged previously.

## Results

### HsGS inactivation by ONOO^−^ and analysis of amino acid nitro-oxidative modifications

**Figure 1:**
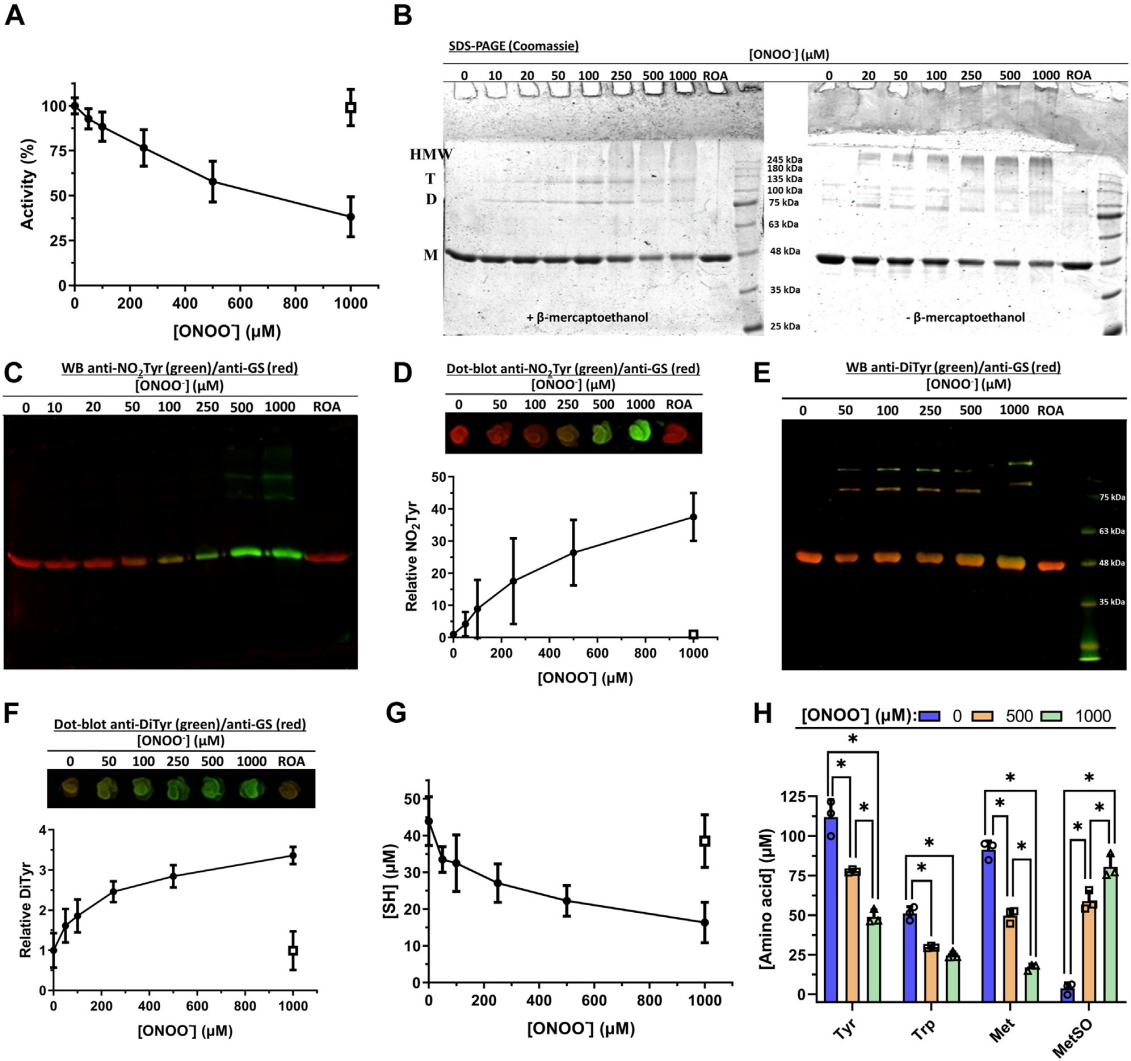
**Inactivation of HsGS by ONOO**^**−**^**and analysis of the main oxidative modifications.** HsGS (0.2 mg ml^−1^, 4.62 μM monomer) was exposed to 50 to 1000 μM ONOO^−^ in KPi buffer pH 7.3 containing 0.1 M KCl and 0.1 mM DTPA. Aliquots were then taken from the samples for the different studies. *A*, relative HsGS activity, expressed as % of the untreated sample (100% activity = 61.4 U/mg) (n = 18). *B*, SDS-PAGE analysis under both reducing and nonreducing conditions. *C*, NO_2_Tyr detection by western blot; anti-NO_2_Tyr reactivity is shown in *green*, anti-GS is shown in *red*. *D*, relative NO_2_Tyr levels, determined through densitometric quantification of anti-NO_2_Tyr dot blots as detailed in Experimental procedures. The anti-NO_2_Tyr/anti-GS ratio for each sample is expressed as relative to the untreated condition (n = 9). *E*, DiTyr detection by western blot; anti-DiTyr reactivity is shown in *green*, anti-GS is shown in *red*. *F*, relative DiTyr levels determined by dot blot (n = 6). *G*, thiol quantification by reaction with DTNB (n = 9). In panels *A*, *D*, *F*, and *G*, the *open squares* represent the reverse order addition (ROA) of ONOO^−^. *H*, amino acid content in total hydrolysates of HsGS (0.4 mg ml^−1^) exposed to ONOO^−^ (500–1000 μM) was measured by fluorescent detection of amino acid-OPA conjugates by UHPLC (n = 3). Data are represented as mean ± sd. ∗*p* ˂ 0.05 by one-way ANOVA analysis with Tukey *post hoc* testing. D, dimer; DTNB, 5,5′-dithiobis(2-nitrobenzoic acid); GS, glutamine synthetase; HMW, higher molecular weight species; M, monomer; OPA, *o*-phthaldialdehyde; T, trimer; UHPLC, ultra high performance liquid chromatography.

Pure HsGS was exposed to ONOO^−^ (50–1000 μM, added as a single bolus) for 5 min or less, with aliquots taken for assessment of residual activity (γ-glutamyl transferase assay) and protein modification (Fig. 1). ONOO^−^ caused a dose-dependent loss of HsGS activity, with a loss of ∼60% detected with 1000 μM ONOO^−^ (Fig. 1*A*). Inactivation was also assessed by the ATP-dependent synthesis of GlnNHOH from glutamate and NH_2_OH, with this yielding similar data (Fig. S2). SDS-PAGE analysis revealed that ONOO^−^ at 10 to 20 μM, or higher, induced the formation of both reducible (disulfide) and nonreducible (nondisulfide) cross-links (dimers, trimers, and higher mass material), resulting in a noticeable decrease in the amount of protein monomer (band at 43 kDa) (Figs. 1*B* and S3).

Immunoblot and dot blot analysis showed a significant increase in the anti-NO_2_Tyr (Fig. 1, *C* and *D*) and anti-DiTyr (Fig. 1, *E* and *F*) immunoreactivity, demonstrating that Tyr residues in HsGS are readily nitrated and oxidized by ONOO^−^. Intense anti-NO_2_Tyr immunoreactivity was detected for the monomer band and also from the bands assigned to cross-linked species at higher ONOO^−^ concentrations (Fig. 1*C*). DiTyr immunoreactivity was detected for the monomer bands, implying the formation of intramonomeric DiTyr. The cross-linked bands, and especially the trimer, also showed significant reactivity (Fig. 1*E*), suggesting that those nonreducible cross-links arise (at least in part) as a consequence of intermolecular DiTyr formation.

In agreement with the detection of reducible cross-links (presumed to be due to disulfides), ONOO^−^ caused a marked consumption of reduced sulfhydryl (-SH) groups in HsGS (Fig. 1*G*). Thus, exposure to 50 μM ONOO^−^ caused a loss of ∼10 μM –SH (2 Cys/HsGS monomer), and ∼28 μM –SH (6 Cys/HsGS monomer) were oxidized with 1000 μM ONOO^−^. Together, these data indicate that exposure of HsGS to excess ONOO^−^ (from ∼10:1–200:1 ONOO^−^:HsGS monomer) caused a dose-dependent loss of HsGS activity and concurrent nitro-oxidative modification of Tyr and Cys residues.

Further data on the amino acids in HsGS that are modified by ONOO^−^ was obtained by subjecting protein hydrolysates to ultra high performance liquid chromatography (UHPLC) analysis for parent amino acid quantification. Treatment of HsGS (0.4 mg ml^−1^) with 500 and 1000 μM ONOO^−^ resulted in a significant loss of Tyr, Trp, and Met residues, but no significant changes to the other native amino acids were detected (Figs. 1*H* and S4). Concurrent with these losses, a significant increase in the main oxidation product of Met, methionine sulfoxide (MetSO), was observed (Figs. 1*H* and S4). These data therefore indicate that Tyr, Trp, Met, and Cys are the major amino acids modified at quantitatively relevant levels by ONOO^−^.

Data on the sites of these modifications induced by ONOO^−^ was obtained by treating native and modified HsGS with trypsin (to release peptides) and subsequent nLC-MS/MS analysis (*i.e.*, peptide mass mapping). For all of the samples examined, a sequence coverage >80 % was obtained (Table S2). Among the residues targeted by oxidants, the following percentages were covered by the peptide mapping analysis: 80% of the Tyr residues (12/15), 71% of the Trp residues (5/7), 69% of the Met residues (9/13), and 91% of the Cys residues (10/11, alkylated). Multiple Tyr residues were detected as nitrated or oxidized (to 3-hydroxytyrosine, DOPA) species, together with limited numbers of nitrated Trp residues and oxidized Met residues. A MS1-filtering approach was applied, using Skyline, to allow comparison of the peak areas of each native and modified peptide and determine which modifications were the most abundant (53) (Fig. S5). Nine Tyr residues were detected as nitrated in ONOO^−^-exposed HsGS (plus the Tyr residue of the His-tag), and five of these were found as oxidized residues. Among these, the highest levels of nitration (>30%) were determined for Tyr185, Tyr269, Tyr288, and Tyr336 for HsGS exposed to 500 μM ONOO^−^. Three Trp residues were detected as nitrated but at a lower level than for the Tyr residues. Seven Met residues were detected as MetSO; for most of these, the levels of modification were high, though some of this oxidation may have occurred during sample processing, as significant levels were also detected in the controls, unlike the other nitrated and oxidized species. These data indicate that Tyr, Trp, and Met residues present at multiple different sites within HsGS can be modified by ONOO^−^ and that modification is not localized to specific sites or regions of the protein structure.

### Tyrosine modification in HsGS by ONOO^−^: nitration and dimerization

**Figure 2:**
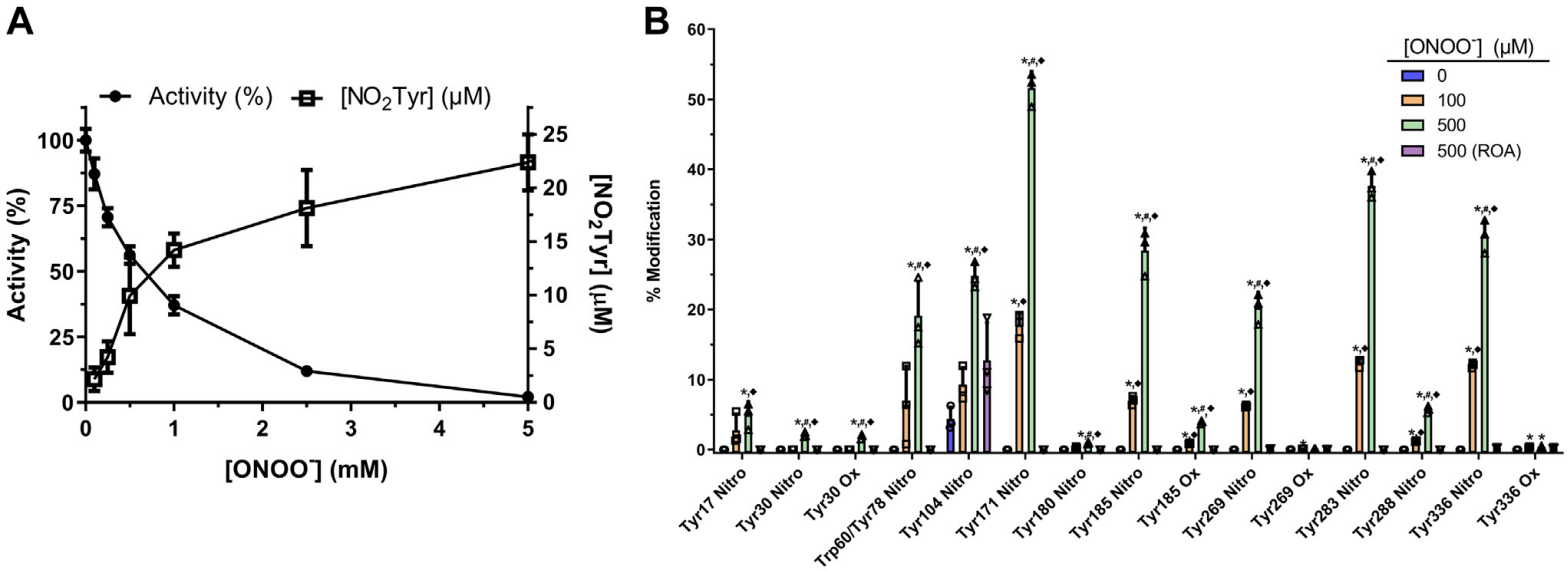
**Quantitative and semiquantitative analysis of HsGS tyrosine nitration by ONOO**^**−**^**.***A*, HsGS (0.2 mg ml^−1^) was treated with 0.1 to 5.0 mM ONOO^−^ in phosphate buffer; aliquots were taken for activity assessment (100% = 61.5 U/mg) (*closed circles*, n = 9), and then protein was precipitated with ice-cold acetone. The pellet was dissolved in 6 M guanidine hydrochloride (1/5 of the original volume), alkalinized with NaOH to pH ≥ 10, and NO_2_Tyr was quantified spectrophotometrically at 430 nm (*open squares*, n = 9). *B*, after exposing HsGS to 100 or 500 μM ONOO^−^, protein was subjected to trypsin digestion and analyzed by MRM-HPLC-MS/MS for targeted detection of specific HsGS peptides (native and modified). Relative levels of modification of each residue were estimated from the peak areas of the detected peptides (n = 3). Data are shown as mean ± sd. ∗*p* ˂ 0.05 (*versus* 0 μM ONOO^−^), ^#^*p* ˂ 0.05 (*versus* 100 μM ONOO^−^), ^♦^*p* ˂ 0.05 (*versus* ROA), by one-way ANOVA analysis with Tukey *post hoc* testing. Trp60/Tyr78 indicates that the transitions detected for the peptide containing residues Trp60 and Tyr78 did not contain enough sequence information to disclose between the two nitration possibilities. MRM, Multiple Reaction Monitoring.

To obtain quantitative data on total NO_2_Tyr levels, HsGS was exposed to higher ONOO^−^ concentrations (0.1–5 mM), enzyme activity was measured, and NO_2_Tyr was quantified spectrophotometrically at 430 nm. Complete HsGS inactivation was obtained with ONOO^−^ concentrations as high as 5 mM (Fig. 2*A*), accompanied by an increase in NO_2_Tyr concentrations from ∼3 μM (at 0.1 mM ONOO^−^) to ∼22 μM (at 5 mM ONOO^−^); the latter corresponds to ∼5 NO_2_Tyr residues per HsGS monomer (Fig. 2*A*). To complement the MS/MS analysis presented above, HsGS samples exposed to ONOO^−^ (50, 250, 500 and 1000 μM) were digested with trypsin and analyzed for NO_2_Tyr-containing peptides, through the detection of NO_2_Tyr-immonium ions (54). This approach identified several of the nitration sites reported above, as well as a previously nondetected nitration site at Tyr171 (Table S1). Thus, the two approaches identified a total of 10 Tyr residues as nitrated species together with the other modified residues described above. To better characterize the Tyr nitration events, a Multiple Reaction Monitoring (MRM) method was developed from the spectra of all the modified peptides (and their native counterparts) to allow a semiquantitative analysis of the main oxidative events under different experimental conditions (Fig. 2*B*). As seen with the MS1 filtering approach (Fig. S5), MRM analysis revealed the presence of preferential nitration targets, with Tyr171 identified as a major nitration site in HsGS together with Tyr283 and that Tyr288 was not as readily nitrated as the MS1 filtering approach suggested. Thus, this approach indicates that the main targets for nitration are Tyr171, Tyr185, Tyr269, Tyr283, and Tyr336. The extents of nitration at these sites is consistent with the loss of HsGS activity (*i.e.*, are high enough to have a quantitatively relevant effect). It should be noted that the values obtained by this method represents an estimation of the extent of modification, and specific values need to be taken with care, as the intensity with which each peptide is detected varies considerably and particularly for the less intense ions. It also needs to be borne in mind that modified and native peptides may ionize with different efficiencies, potentially skewing the data. Despite these considerations, this approach can be a useful tool for distinguishing major (*e.g.*, Tyr171, Tyr336) from minor (*e.g.*, Tyr17, Tyr180) nitration sites.

In addition to the conversion of Tyr residues to NO_2_Tyr and DOPA (see above), immunoblot and dot blot analysis revealed the presence of DiTyr cross-links between HsGS subunits. Through performing trypsin digestion of ONOO^−^-treated HsGS in H_2_^18^O *versus* H_2_^16^O, which results in C-terminal labeling of the new peptide carboxyl groups with either ^18^O or ^16^O and subsequent nLC-MS/MS analysis (55, 56), five different DiTyr cross-links were detected (Fig. S6) between Tyr288-Tyr269, Tyr269-Tyr269, Tyr288-Tyr283, Tyr336-Tyr336, and Tyr30-Tyr180. It is noteworthy that all of these residues were also detected as nitrated species, confirming that nitration and dimerization induced by ONOO^−^-derived radicals occurs *via* a common intermediate tyrosyl radical. No other types of cross-links (Tyr-Trp, Trp-Trp, Lys-Tyr, Lys-His, or His-His) were identified.

### HsGS inactivation by ONOO^−^ is mediated by one-electron oxidants

**Figure 3:**
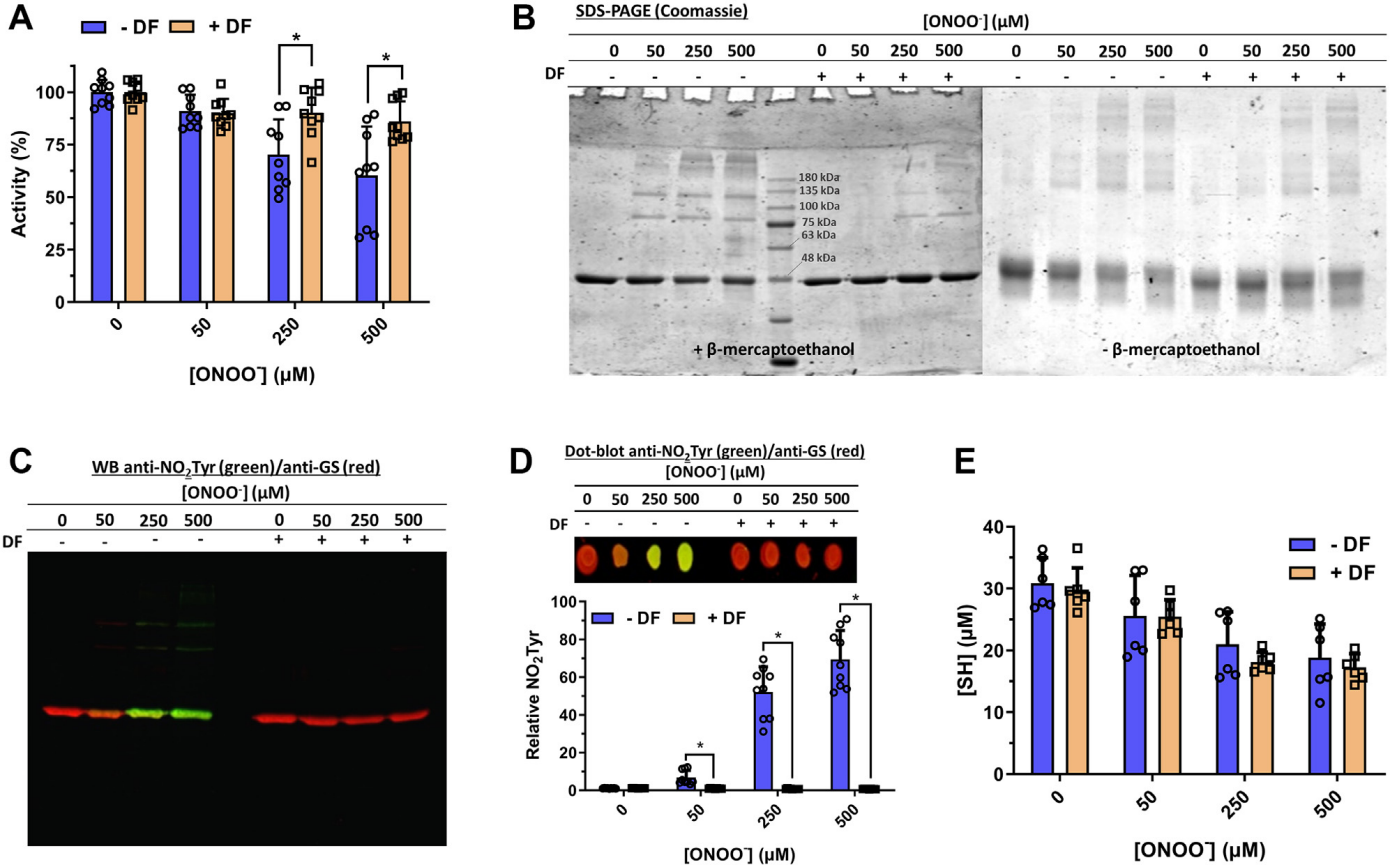
**Effect of desferrioxamine on HsGS inactivation and oxidative-modification by ONOO**^**−**^**.** HsGS (0.2 mg ml^−1^) was exposed to ONOO^−^ (50–500 μM) in KPi buffer pH 7.3 either in the absence (−DF) or presence (+DF) of 1 mM desferrioxamine. After treatment, aliquots were taken for: (*A*) activity measurements (n = 9), expressed as relative to each control (100% = 59.9 and 55.0 U/mg, −DF and +DF respectively), (*B*) SDS-PAGE analysis under both reducing and nonreducing conditions, (*C*) NO_2_Tyr detection by western blot and (*D*) dot blot (n = 9) and (*E*) thiol quantification (n = 6). In panels (*C* and *D*), NO_2_Tyr staining is shown in *green*, and GS staining in *red*. Data are represented as mean ± sd. ∗*p* ˂ 0.05 (−DF *versus* +DF) by unpaired t tests analysis. DF, desferrioxamine; GS, glutamine synthetase.

In the experiments described above, HsGS modification might arise from direct two-electron oxidation (of Cys, Met, or Trp residues) or *via* one-electron oxidations mediated by ONOO^−^-derived radicals (at Cys, Met, Trp, or Tyr residues). To examine which process mediates enzyme inactivation, HsGS was exposed to ONOO^−^ in the absence or presence of 1 mM desferrioxamine (DF), an iron chelator and radical scavenger (57, 58, 59). HsGS inactivation by higher concentrations of ONOO^−^ was largely prevented by DF (Fig. 3*A*), accompanied by a marked decrease in nonreducible cross-links but only a modest decrease in reducible (disulfide) cross-links (Fig. 3*B*). Consistent with these observations, DF completely inhibited ONOO^−^-mediated HsGS Tyr nitration (Fig. 3, *C* and *D*) but had no effect on the loss of Cys residues (Fig. 3*E*). This suggests that ONOO^−^-mediated HsGS inactivation occurs primarily *via* a mechanism involving one-electron oxidation and modifications induced by ^•^NO_2_ and ^•^OH, rather than *via* direct oxidations mediated by ONOO^−^/ONOOH.

**Figure 4:**
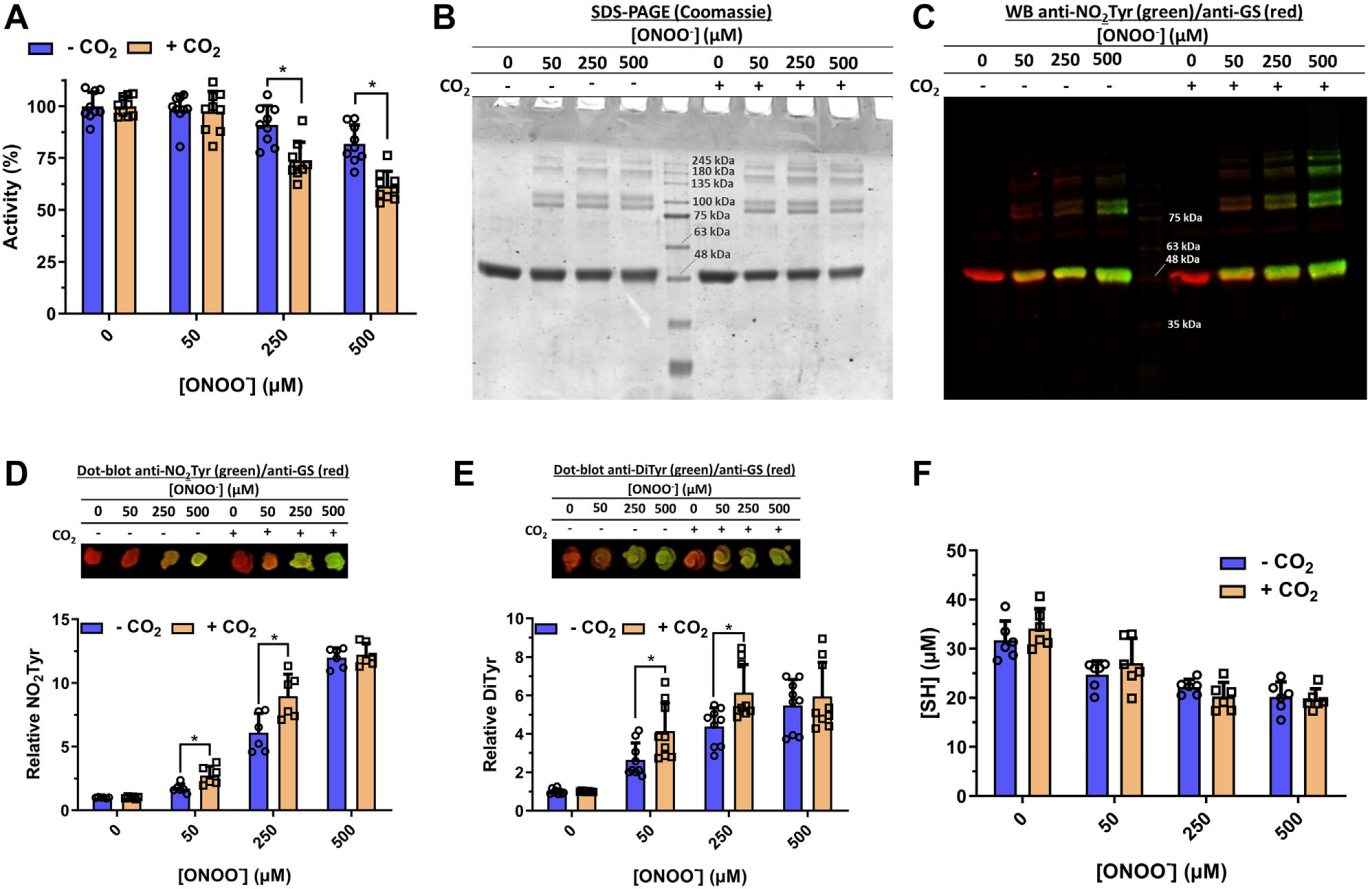
**CO**_**2**_**enhances ONOO**^**−**^**-mediated oxidative inactivation of HsGS.** To assess the effect of CO_2_, HsGS (0.2 mg ml^−1^) was exposed to ONOO^−^ (50–500 μM) in KPi buffer pH 7.3 without (−CO_2_) or with (+CO_2_) 25 mM NaHCO_3_. After ONOO^−^ exposure, samples were characterized by (*A*) activity measurements (n = 9), expressed as relative to each control (100 % = 51.3 U/mg and 50.0 U/mg, −CO_2_ and +CO_2_ respectively), (*B*) SDS-PAGE analysis under reducing conditions, (*C*) NO_2_Tyr detection by western blot and (*D*) dot blot (n = 6), (*E*) DiTyr detection by dot blot (n = 9), and (*F*) thiol quantification (n = 6). In panels (*C–E*), NO_2_Tyr/DiTyr staining is shown in *green*, and GS staining in *red*. Data are represented as mean ± sd. ∗*p* ˂ 0.05 (−CO_2_*versus* +CO_2_) by unpaired t tests analysis. GS, glutamine synthetase.

The mechanism of HsGS inactivation was also probed by the inclusion of 25 mM sodium bicarbonate (NaHCO_3_), which is in equilibrium with ∼1.2 mM CO_2_, in the reaction mixture, competing with direct protein-ONOO^−^ reactions and enhancing the selectivity and radical yield that arise due to ONOO^−^ decay. In the presence of CO_2_, high concentrations of ONOO^−^ caused a more pronounced inactivation of HsGS (Fig. 4*A*) and increased the formation of nonreducible protein cross-links (Fig. 4*B*), Tyr nitration (Fig. 4, *C* and *D*), and Tyr dimerization to DiTyr (Fig. 4*E*). Oxidation of Cys residues was not affected significantly by CO_2_ (Fig. 4*F*), suggesting that such oxidation occurs either through direct oxidation or *via* ONOO^−^-derived radicals. Together, these data indicate that oxidative modifications such as Tyr nitration and dimerization appear to be mediating the loss of activity, while Cys oxidation does not directly impact on enzyme activity.

**Figure 5:**
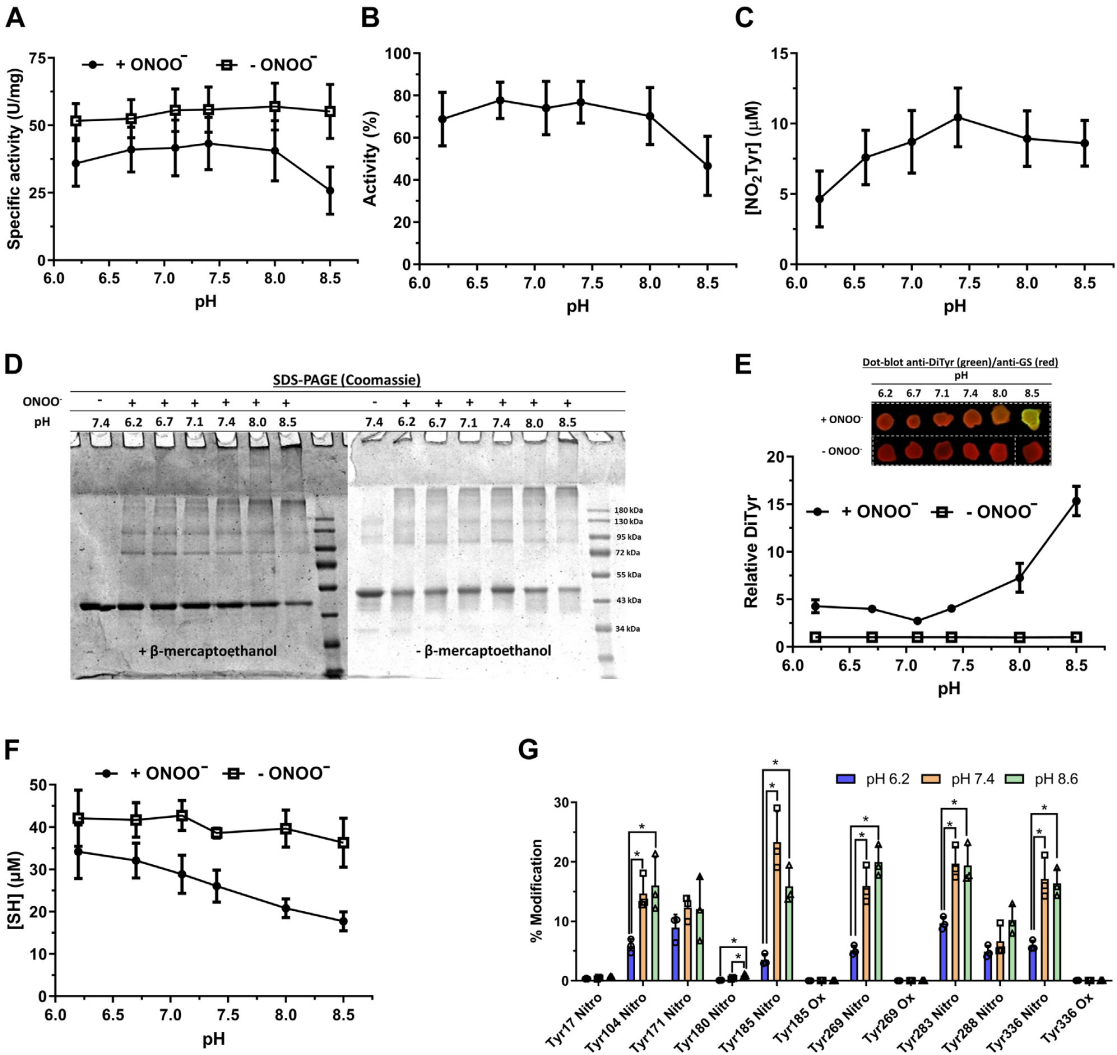
**pH dependency of HsGS inactivation by ONOO**^**−**^**.** Exposure of HsGS (0.2 mg ml^−1^) to a single bolus of 0.5 mM ONOO^−^ was done in KPi buffer solutions of varying pH values (6.2–8.6) to analyze how modulating ONOO^−^ reactivity influences on HsGS inactivation. *A*, specific HsGS activity was measured at every pH both for treated or untreated samples (n = 12). *B*, relative activity of treated samples at different pH values, expressed as % of their respective pH control (see panel *A*) (n = 12). *C*, NO_2_Tyr quantification in treated samples through spectrophotometric measurements (n = 9). *D*, reducing and nonreducing SDS-PAGE analysis of ONOO^−^-exposed HsGS at different pH values. *E*, dot blot assessment of relative DiTyr levels under varying pH-values; DiTyr levels are expressed as relative to each control (n = 3). The *white dashed lines* in the representative dot blot correspond to the spliced borders made from the primary scanned image in order to achieve a more suitable signal display in the Figure. *F*, thiol quantification at the different pH values for either treated or untreated HsGS samples (n = 6). *G*, MRM-HPLC-MS/MS relative quantification of the modification degree of Tyr residues by ONOO^−^ at three selected pH values (n = 3). Data are represented as mean ± sd. ∗*p* ˂ 0.05 by one-way ANOVA analysis with Tukey *post hoc* testing. MRM, Multiple Reaction Monitoring.

The dependence of these mechanisms with pH was also evaluated. For this, HsGS was exposed to a single 500 μM bolus of ONOO^−^ in KPi buffer at pH values over the range 6.2 to 8.5. The control specific activity (without ONOO^−^) was not affected by incubation over this pH range (Fig. 5*A*), with the final activity assay carried out at pH 6.8 in each case. The loss of activity induced by ONOO^−^, which showed minimal variation over the range 6.2 to 7.4, tended to be greater at pH values > 7.4 (Fig. 5*B*). SDS-PAGE analysis of the treated samples revealed that cross-link formation, and especially nonreducible processes, increased at higher pH values (Fig. 5*D*), while Tyr nitration (as determined by spectrophotometry and dot blot analysis) was at a maximum at pH ∼7.4, with decreases detected at both more acidic or alkaline pHs (Fig. 5*C*). Dot blot analysis also showed that DiTyr formation did not vary significantly over the range 6.2 to 7.4 but increased notably at pH values > 7.4 (Fig. 5*E*). The extent of Cys oxidation also increased at higher pH values (Fig. 5*F*). MRM-MS/MS analysis of the relative levels of NO_2_Tyr at three selected pH values (6.2, 7.4 and 8.6) revealed that, for most residues, nitration was significantly higher at pH 7.4 than at pH 6.2 (by ∼2-fold), but it showed no significant differences between pH 7.4 and 8.6, with the exception of Tyr180 which is a minor target (Fig. 5*G*).

Together, these data indicate that oxidative inactivation of HsGS is a complex process that cannot be explained by a single type of modification at a single residue (*cf.* a similar conclusion reached with (60)). Thus, the greatest loss of activity was detected at pH values where Tyr nitration was not maximal, suggesting that this processes is not directly causal. In contrast, DiTyr formation and Cys oxidation were maximal at pH values at which the greatest inactivation was detected, raising the possibility that such modifications contribute to the loss of activity mediated by ONOO^−^.

### Inactivation of five HsGS Tyr→Phe mutants by ONOO^−^

**Figure 6:**
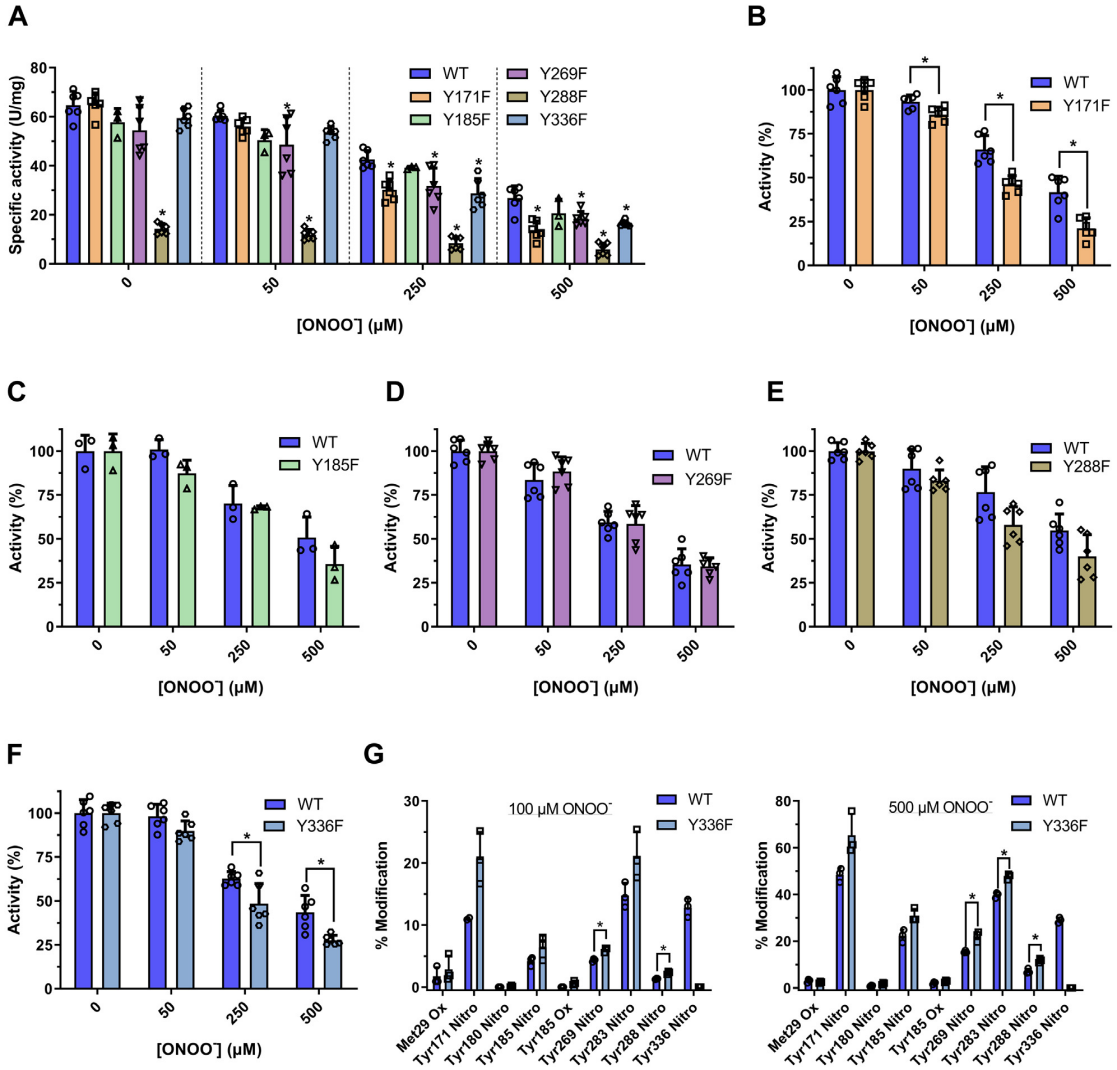
**Inactivation of five Tyr→Phe HsGS mutants by ONOO**^**−**^**.** The five HsGS Tyr→Phe mutants purified (0.2 mg ml^−1^) were exposed to ONOO^−^ (50–500 μM) and their inactivation was compared to the WT enzyme. *A*, specific activities measured for HsGS WT and the five HsGS mutants treated with ONOO^−^(n = 3–6). *B–F*, comparison of HsGS activities expressed as relative to each control (untreated enzyme, see panel *A*). *G*, the modification degree of certain specific residues, as estimated by MRM-HPLC-MS/MS analysis, was determined for the Y336F mutant exposed to 100 and 500 μM ONOO^−^ and compared to the WT HsGS. ∗*p* ˂ 0.05 (*versus* WT) by one-way ANOVA analysis with Tukey *post hoc* (*A*) or *p* ˂ 0.05 (mutant *versus* WT) by unpaired t tests analysis (*B–G*). MRM, Multiple Reaction Monitoring.

In order to assess the specific role of some of the main Tyr residues that undergo significant levels of nitration and oxidation, five different Tyr(Y)→Phe(F) mutants of HsGS were generated (Y171F, Y185F, Y269F, Y288F, and Y336F). These residues were selected on the basis of their extent of nitration or involvement in DiTyr cross-links. Each variant was then exposed to ONOO^−^ in KPi buffer alongside the WT enzyme to compare the oxidative inactivation processes. Under control conditions, the Y171F, Y185F, and Y336F mutants showed specific activities that were not significantly different to the WT enzyme. The Y269F mutant had a slightly decreased activity, while the Y288F variant showed a markedly decrease (∼80%) (Fig. 6*A*). All the mutants were susceptible to inactivation by ONOO^−^ (Fig. 6*A*). When comparing each of the variants against WT HsGS in terms of relative activity (normalized with respect to the specific activity of each untreated sample), a higher degree of inactivation was seen for the Y171F and Y336F mutants, while no differences were observed for the other variants (Fig. 6, *B*–*F*). Interestingly, both Tyr171 and Tyr336 were found to be the main targets of nitration in the MRM experiments, so the fact that these mutants were more susceptible to inactivation suggests that their nitration does not represent a major route to HsGS inactivation under the conditions used here.

As the lack of a particular target would be expected to increase the extent of modification at other targets (that may be linked to oxidative inactivation), this was examined. Treatment of the Y336F mutant with 500 μM ONOO^−^ led to a significant increase in nitration at Tyr269, Tyr283, and Tyr288 (Fig. 6*G*), raising the possibility that nitration at one or more of these sites is related to the loss of function. As the Y269F mutant was inactivated to the same extent as the control, it is unlikely that nitration at this site results in inactivation. No data was obtained with regard to Tyr283, as no mutant for this residue was available. Regarding Tyr288, although it was nitrated to a less extent than the other Tyr residues that were mutated, its nitration may be of relevance as this Tyr→Phe mutation had a profound effect on enzyme activity. Thus, modification of Tyr288 by nitration may have a similar effect, even though its modification is not quantitatively the largest. Other comparative biochemical characterizations of the oxidative modifications of the mutants showed no great differences (Figs. S7 and S8). Only the Y288F mutant showed a higher susceptibility to Cys oxidation, as seen both quantitatively and by nonreducing SDS-PAGE analysis. The relative levels of NO_2_Tyr for the Y171F species were significantly decreased as assessed by dot blot analysis and also (to a lesser extent) by immunoblot analysis, something that was also observed for the Y336F mutant. Nonreducible cross-linking appeared to be decreased for the Y269F and Y288F mutants, in agreement with their involvement in three of the DiTyr cross-links reported above.

Altogether, these data obtained from the Tyr→Phe mutants suggests that oxidative inactivation of HsGS is not mediated exclusively by modification at any of these residues. The absence of any of these residues appears to redirect damage to other targets, something that in particular cases (Y171F, Y336F) leads to a greater loss of activity. Furthermore, these studies reveal the importance of Tyr288 in maintaining the normal activity of HsGS.

### HsGS aggregation induced by ONOO^−^

The nonreducible SDS-PAGE analyses reported above revealed that ONOO^−^ induces the formation of species with molecular masses high enough to impede migration into the gels employed. In some cases, this effect was also seen under reducing conditions and for stock solutions kept for long periods. These observations prompted an examination of the propensity of HsGS to undergo aggregation and the relevance of this process to oxidative inactivation.

**Figure 7:**
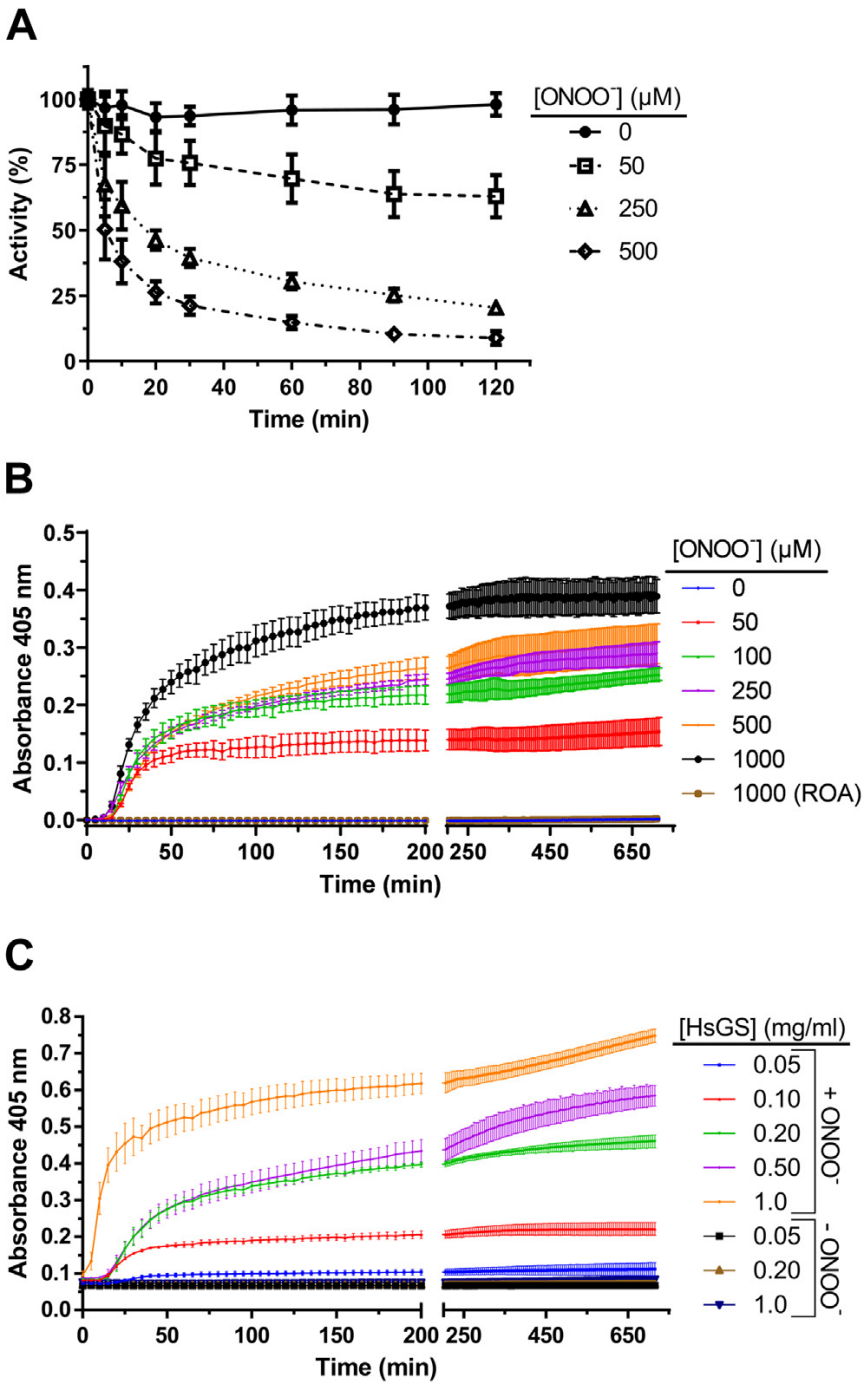
**ONOO**^**−**^**-induced aggregation of HsGS.***A*, time-dependent loss of HsGS activity after exposing the protein (0.2 mg ml^−1^) to a single bolus of ONOO^−^ (50–500 μM) and incubating it for 120 min at 37 °C. Aliquots were taken at the indicated times for activity measurements; values are expressed as relative to the activity of the untreated control at time 0 (100 % = 58.7 U/mg, n = 6). *B*, the aggregation of HsGS (0.2 mg ml^−1^) after exposure to a single bolus of ONOO^−^ (50–1000 μM) was followed by turbidity measurements over a 12 h period (n = 3). *C*, aggregation of HsGS (0.05–1.0 mg ml^−1^) over 12 h after being exposed to 500 μM ONOO^−^ (n = 3). Data are shown as mean ± sd.

Loss of HsGS activity was measured over a 2 h period after exposure to a single ONOO^−^ bolus in KPi buffer, pH 7.3, at 21 °C for 5 min, followed by incubation at 37 °C with slow shaking, with aliquots removed at different time points to assess HsGS activity (as described above). While the loss of HsGS activity at 5 min was comparable to the values reported above, a further significant decrease in activity was detected with increasing incubation time for all the treated samples (Fig. 7*A*). For instance, with 50 μM ONOO^−^ addition, only a 10% loss of HsGS activity was detected at 5 min, but by 2 h, this had increased to ∼40%. Even greater decreases in HsGS activity were observed during the 2 h incubation period for samples exposed to 250 and 500 μM ONOO^−^. As ONOO^−^ decays completely in a matter of seconds under these conditions, the loss of HsGS activity observed during the 2 h incubation period is likely to be a consequence of slow secondary processes arising from the oxidative modifications induced by ONOO^−^, directly or indirectly (through its derived radicals) during its decomposition. Noncovalent aggregation of oxidized HsGS may be an explanation for this observation. To further examine this phenomenon, aggregate formation was assessed by turbidity measurements over 12 h, in a 96-well plate, after addition of a single bolus of ONOO^−^. When a fixed HsGS concentration (0.2 mg ml^−1^) was exposed to different ONOO^−^ concentrations, an initial lag phase of 15 to 20 min was observed followed by a significant increase in turbidity over 30 to 60 min. This was followed by a much less-pronounced increase during the next 1 h and finally a plateauing effect. Both the initial slope and the amplitude of the curves increased with higher ONOO^−^ concentrations (Fig. 7*B*). Similar curves were obtained when a constant concentration of ONOO^−^ (500 μM) was added to increasing concentrations of HsGS (0.05–1.0 mg ml^−1^); in this case, the slope and amplitude tended to increase as the protein concentration, although following different behaviors (Fig. 7*C*). These results indicate that ONOO^−^ is capable of promoting HsGS aggregation *via* initial oxidative modification, besides causing its inactivation, with this being the time-dependent process responsible for major losses of HsGS activity after ONOO^−^ exposure.

### Thiol oxidation triggers HsGS aggregation induced by oxidants

**Figure 8:**
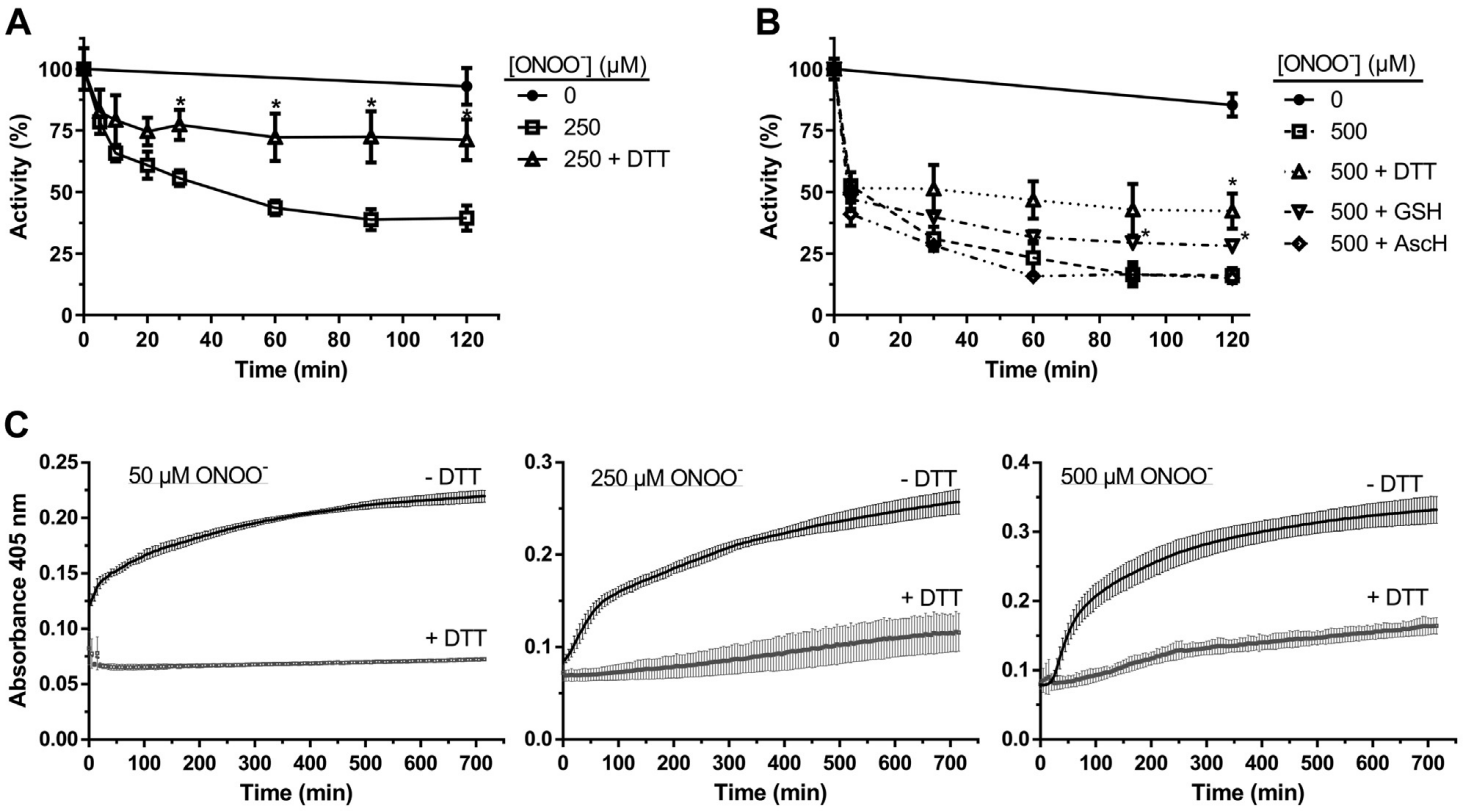
**Reduction of oxidized thiols after ONOO**^**−**^**treatment inhibits HsGS aggregation.***A*, HsGS (0.2 mg ml^−1^) was exposed to ONOO^−^ in KPi buffer and after a 1 min incubation at 21 °C, 10 mM DTT was added to reduce oxidized thiols. After that, the protein was incubated at 37 °C for 2 h, and aliquots were taken for activity measurements at different time points (100% = 62.2 U/mg). *B*, same as (*A*) but with the inclusion of other reductants, namely, glutathione (GSH) and ascorbic acid (AscH). *C*, alternatively, after reduction of oxidized protein thiols with DTT, HsGS was placed in a 96-well plate and incubated at 37 °C for 12 h for turbidity measurements. Data are shown as mean ± sd (n = 3). ∗*p* ˂ 0.05 (*versus* +ONOO^−^ only) by unpaired t test analysis.

As previous studies had reported the aggregation of sheep brain GS as a consequence of exposure to thiol alkylating agents (*e.g.*, *N*-ethylmaleimide (61, 62)), the possibility that Cys modification could be responsible for inducing HsGS aggregation after ONOO^−^ exposure was examined. HsGS was exposed to a single bolus of ONOO^−^ (250–500 μM), 10 mM DTT was added after a 1 min incubation at 21 °C (to allow complete decomposition of ONOO^−^ and its derived radicals), and finally the protein was incubated at 37 °C for 2 h with slow shaking. Aliquots were taken at different times for activity measurements. Once again, a significant loss of HsGS activity was observed over the incubation period after ONOO^−^ addition; however, this loss was largely prevented when the enzyme was treated with DTT after being oxidized by ONOO^−^ (Fig. 8, *A* and *B*). A similar result was obtained when GSH was used instead of DTT as the thiol reducing agent. No protection was afforded by ascorbate, a one-electron reductant (Fig. 8*B*). In agreement with these data, HsGS aggregation was significantly prevented when, after addition of ONOO^−^, the protein was incubated at 37 °C in the presence of DTT (Fig. 8*C*). These data suggest that Cys oxidation induced by ONOO^−^ can mediate HsGS aggregation, provoking an additional loss of protein activity, above that induced by the rapid oxidative modification of key amino acids by ONOO^−^.

**Figure 9:**
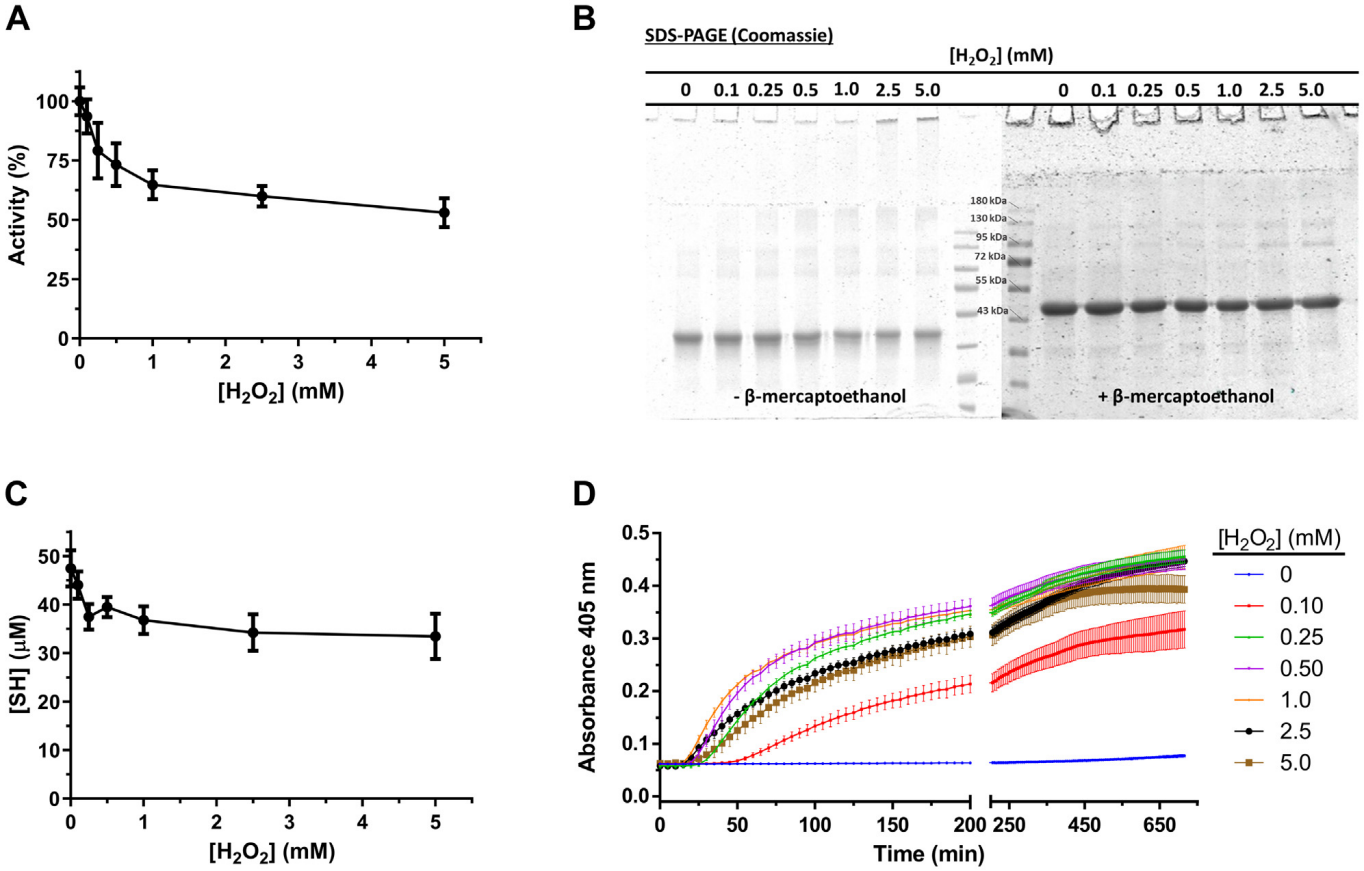
**Loss of HsGS function by H**_**2**_**O**_**2**_**-dependent thiol oxidation and protein aggregation.** Oxidation of HsGS by H_2_O_2_ was carried out by incubating 0.2 mg ml^−1^protein in 100 mM KPi buffer pH 7.3 containing 0.1 M KCl and 0.1 mM DTPA with H_2_O_2_ (0.1–5 mM) at 37 °C for 1 h. Reactions were then stopped by addition of 50 nM catalase and aliquots were taken for: (*A*) activity measurements (100% = 64.5 U/mg, n = 9), (*B*) reducing and nonreducing SDS-PAGE analysis, and (*C*) thiol quantification (n = 3). *D*, H_2_O_2_-induced aggregation was studied by directly incubating 0.2 mg ml^-1^ HsGS in a 96-well plate with the indicated amounts of H_2_O_2_ at 37 °C for 12 h with turbidity measurements every 5 min (n = 3). Data are shown as mean ± sd.

To further examine this hypothesis, the ability of two alternative two-electron oxidants, H_2_O_2_ and diamide, which mainly target Cys residues, to promote HsGS aggregation was evaluated. Incubation of HsGS with H_2_O_2_ (0.1–5 mM) in KPi buffer pH 7.3 for 1 h at 37 °C led to a significant loss of activity (Fig. 9*A*), though this was less pronounced than seen with ONOO^−^. This was accompanied by a significant decrease in thiol content (Fig. 9*C*) and formation of reducible cross-links and high molecular mass aggregates (as detected by SDS-PAGE, which were not detected under reducing conditions (Fig. 9*B*)). H_2_O_2_ also induced HsGS aggregation as evidenced by turbidity measurements (Fig. 9*D*). However, it should be noted that H_2_O_2_ is also capable of oxidizing Met residues, although at slower rates (44). To rule out this possibility, the thiol-specific oxidant diamide was used. Incubation of HsGS with diamide strongly decreased HsGS activity, resulted in loss of Cys residues, and induced protein aggregation in a dose-dependent manner (Fig. S9). Together, these results indicate that Cys oxidation is sufficient to induce HsGS aggregation, with an associated loss of activity.

To investigate whether other one-electron oxidants can also induce aggregation, experiments were carried out using 2,2′-azobis (2-amidinopropane) dihydrochloride (ABAP) which generates peroxyl radicals (ROO^•^) at a defined rate in a time-dependent manner. Incubation of HsGS with ABAP (10 mM, 37 °C in KPi buffer pH 7.3) resulted in complete loss of activity after 120 min, together with the formation of nonreducible cross-links and a fast and marked increase in solution turbidity (Fig. S10). A good correlation was observed between the kinetics of aggregation and inactivation, supporting a connection between these processes. Inclusion of DF (1 mM) in the reaction mixture delayed both the inactivation and aggregation of HsGS induced by ABAP (Fig. S10). Interestingly, inclusion of NO_2_^−^ in the reaction system, which could be oxidized by the peroxyl radicals to yield ^•^NO_2_, had only minor effects, with regards to a delay in aggregation and loss of activity (Fig. S10). Thus, under this condition, the possibility for Tyr nitration to occur (detected by western blot only after 2 h incubation, data not shown) did not increase the loss of HsGS activity nor its aggregation.

### Other factors implied in HsGS aggregation induced by ONOO^−^

To obtain more mechanistic data on the biochemical events that result in HsGS aggregation, the kinetics of aggregation induced by ONOO^−^ were analyzed under different conditions. The presence of CO_2_ enhanced HsGS aggregation (Fig. S11*A*), and considering the data reported above, where thiol oxidation was not affected by CO_2_, this suggests that other amino acid modifications could contribute to aggregation in addition to Cys oxidation. HsGS also showed an increased tendency to aggregate after ONOO^−^ addition at acid and, especially, alkaline pH values, when compared to pH 7.0 to 7.4 (Fig. S11*B*). HsGS showed a significant increase in turbidity over time without ONOO^−^ addition at pH 8.6, suggesting that the stability of this protein is affected by nonoptimal pH conditions. Turbidity studies using the different Tyr→Phe mutants revealed no relevant differences in the ONOO^−^-induced aggregation of the Y171F and Y269F variants and a slight increased tendency to aggregate for the Y185F and Y336F mutants (Fig. S11*C*). The Y288F mutant showed a notably accelerated increase in turbidity after exposure to ONOO^−^, and also in the absence of oxidant, it aggregated to a comparable extent to WT HsGS exposed to 500 μM ONOO^−^ (Fig. S11*C*). This finding support the idea that Tyr288 plays a key role in maintaining HsGS structure and that its nitration/dimerization, although quantitatively minor, might have a strong impact on HsGS function. To this point, data suggests that Cys oxidation induces HsGS aggregation and that only the modified protein undergoes such process, without promoting the aggregation of unmodified HsGS (Fig. 7*B*, the amplitude of the curves is dependent on the amount of oxidant). To examine this hypothesis, the ability of aggregated HsGS to induce the aggregation of native HsGS was assessed (Fig. S11*D*). Different amounts of ONOO^−^-aggregated HsGS (0–20 μg) were unable to induce the aggregation of untreated HsGS (0.2 mg ml^−1^, 200 μl); thus, oxidant-induced aggregation of HsGS is limited to the fraction of Cys-oxidized HsGS molecules.

**Figure 10:**
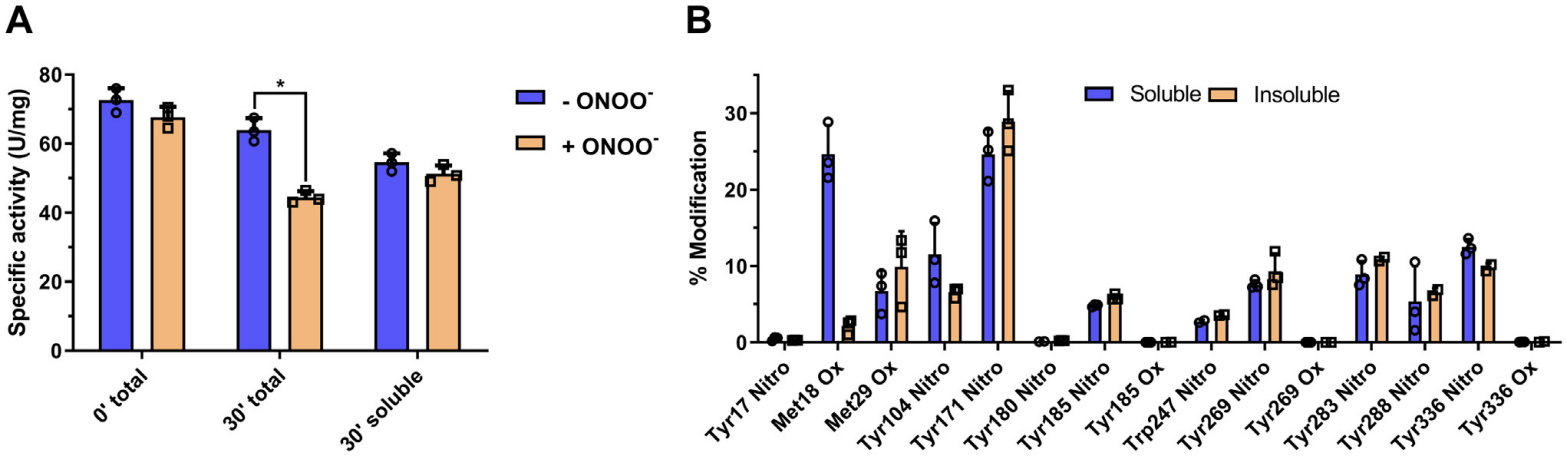
**Characterization of soluble and insoluble protein after ONOO**^**−**^**treatment.** HsGS (0.5 mg ml^−1^) was exposed to a single ONOO^−^ bolus (500 μM), incubated 30 min at 37 °C and after that, insoluble protein was separated by centrifugation at 18,500*g* for 30 min. The supernatants were collected and the remaining protein content was quantified. *A*, HsGS specific activity was measured in the samples immediately after ONOO^−^ addition (0 min total), after the 30 min incubation at 37 °C (30 min total) and in the corresponding supernatants after removal of insoluble protein (30 min soluble). *B*, MRM MS/MS analysis of both soluble and insoluble trypsin-digested HsGS after exposure to ONOO^−^ and incubation at 37 °C for 30 min. Data are shown as mean ± sd (n = 3). ∗*p* ˂ 0.05 (−ONOO^−^*versus* +ONOO^−^) by unpaired t tests analysis. MRM, Multiple Reaction Monitoring.

Finally, to verify that the time-dependent loss of activity is due to aggregation of modified HsGS and loss of soluble protein, HsGS specific activity was measured before and after the removal of insoluble material. For this, HsGS (0.5 mg ml^−1^) was exposed to ONOO^−^ (500 μM), aliquots were immediately taken for activity measurements, and the protein was incubated for 30 min at 37 °C. After incubation, activity in the mixture (both soluble and insoluble protein) was measured, and then the samples were subjected to centrifugation to remove precipitated HsGS. The protein remaining in the supernatant was quantified, and activity of the soluble HsGS was measured. After initial ONOO^−^ exposure (t = 0 min, total), HsGS activity showed no significant decrease with respect to the control; however, 30 min after (t = 30 min, total), the specific activity of the treated sample was significantly decreased with respect to the untreated control (Fig. 10*A*). After removal of insoluble protein, both specific activities were similar, as observed immediately after ONOO^−^ exposure (Fig. 10*A*). This indicates that the time-dependent loss of HsGS activity after exposure to a single bolus of ONOO^−^ is a consequence of the precipitation of aggregated protein. To gain further information with respect to the factors determining this process, both protein pellets and supernatants were subjected to trypsin digestion and MRM analysis by HPLC-MS/MS (as above). No significant differences were observed in regard to the extent of modification of the Tyr residues between the soluble and insoluble fractions, suggesting that their modification is not a primary factor promoting aggregation under these conditions (Fig. 10*B*).

### Effect of MgATP binding on HsGS stability and oxidative-induced aggregation

**Figure 11:**
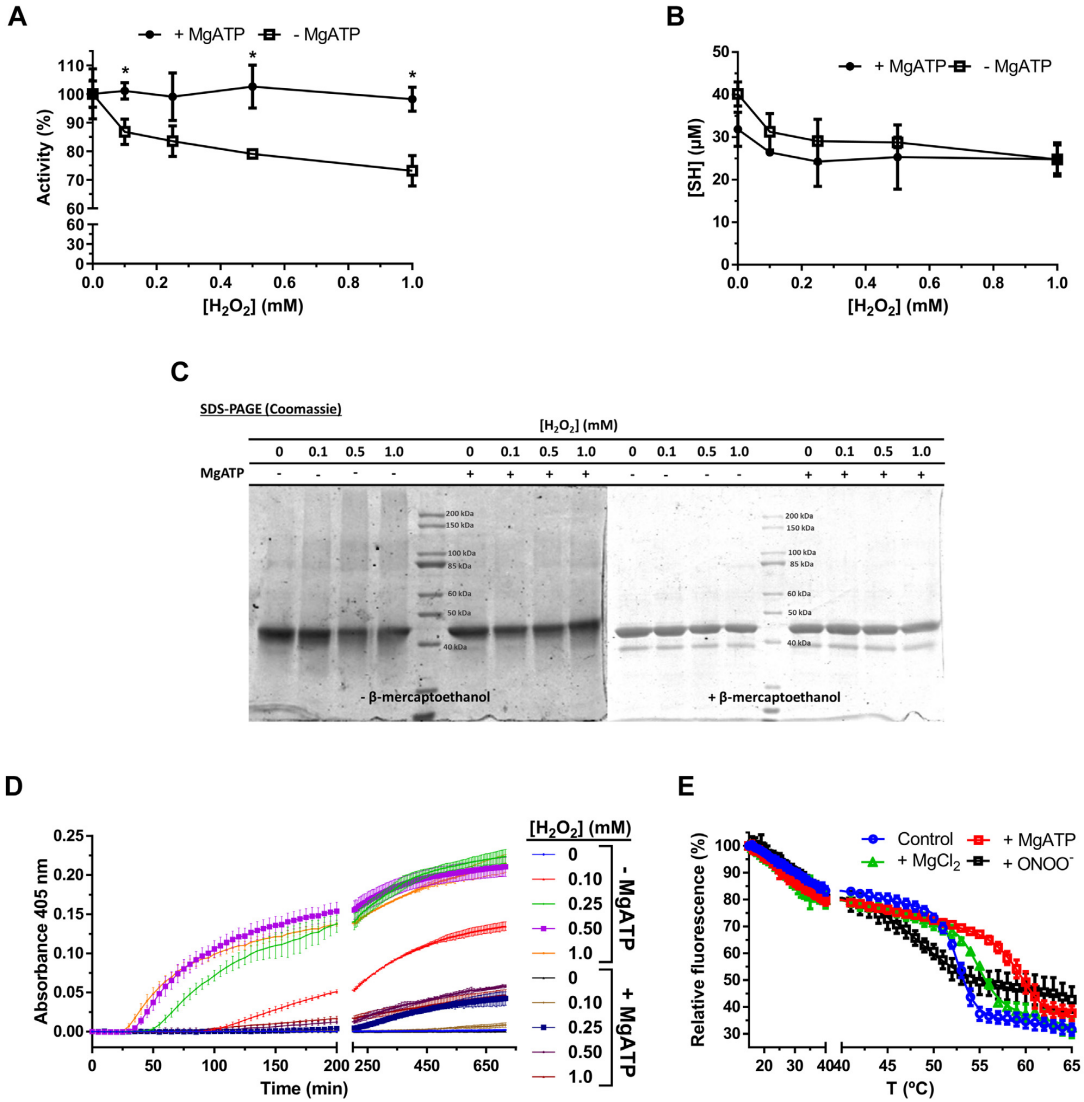
**Effect of MgATP binding on H**_**2**_**O**_**2**_**-induced HsGS aggregation and protein stability.** Treatment of HsGS (0.2 mg ml^−1^) with H_2_O_2_ (0.1–1.0 mM) was carried out for 1 h at 37 °C either in the absence or presence of 10 mM ATP and 20 mM MgCl_2_. After stopping the reactions with catalase, aliquots were taken for: (*A*) activity assays (100 % = 63.4 and 48.7 U/mg, without and with MgATP, respectively), (*B*) thiol quantification, and (*C*) reducing and nonreducing SDS-PAGE analysis. *D*, aggregation was studied by turbidity measurements at 405 nm for 12 h after incubation of HsGS (0.2 mg ml^−1^) with H_2_O_2_ (0.1–1.0 mM) at 37 °C in the absence or presence of 10 mM ATP and 20 mM MgCl_2_. *E*, thermal denaturation of HsGS either alone, bound to MgATP or Mg^2+^, or treated with ONOO^−^, was followed by measuring the decrease of Trp fluorescence (λ_ex_ 295 nm, λ_em_ 335 nm) at increasing temperatures. Data are shown as mean ± sd (n = 3). ∗*p* ˂ 0.05 (−MgATP *versus* +MgATP) by unpaired t tests analysis.

The above data suggest that HsGS aggregation is a consequence of an overall loss of protein stability that can result from oxidant exposure (and probably Cys oxidation), pH values far from physiological, or the presence of point mutations at critical sites (*e.g.*, Y288F). Previous studies have reported that some ligands (*e.g.*, Mg^2+^, Mn^2+^, and adenine nucleotides) can help maintain GS stability and promote its decameric assembly (62, 63, 64), and incubation of mammalian GS with Mg^2+^ and ATP can prevent the loss of activity induced by thiol alkylating agents (64, 65). To test the effects of such ligand, HsGS oxidation, loss of activity, and aggregation induced by H_2_O_2_ was evaluated in the absence or presence of MgATP. Incubation of HsGS with H_2_O_2_ (0.1–1 mM) for 1 h at 37 °C led to a significant decrease in enzyme activity, thiol content, and formation of reducible protein cross-links (as reported above). However, these effects were attenuated when MgATP was present in the mixture (Fig. 11, *A*–*C*), with H_2_O_2_-induced aggregation of HsGS strongly inhibited by MgATP (Fig. 11*D*). To confirm whether MgATP binding increased HsGS stability, thermal denaturation studies were carried out through Trp fluorescence measurements over the temperature range 15 to 65 °C. In the presence of MgATP, the melting curve for HsGS significantly shifted to the right, increasing the Tm value from 52.7 °C ± 0.1 deg for HsGS alone to 60.8 °C ± 1.3 deg for MgATP-bound protein (Fig. 11*E*). In the presence of Mg^2+^ alone, the Tm value was between the above values 55.0 °C ± 0.2 deg. These results suggest that ligand binding not only stabilizes HsGS but can also induce conformational changes that protect critical Cys residues from oxidation, making the protein resistant to aggregation. In contrast to the ligand-binding data, exposure of HsGS to ONOO^−^ caused a change in the shape of the denaturing curve, suggesting that the oxidatively modified protein, expected to bear conformational changes, adopted a different unfolding mechanism. A slightly decreased Tm value of 50.3 °C ± 1.2 deg was also determined, consistent with a less stable proteoform (Fig. 11*E*); this may underlie the predisposition of ONOO^−^-treated HsGS to undergo aggregation. In order to assess the possibility that MgATP binding was not only increasing HsGS stability but also promoting its decameric assembly, gel filtration studies were carried out. Native HsGS (4.0 mg ml^−1^) in phosphate buffer eluted almost entirely as a single peak at ∼16 ml, with a minor fraction at ∼14.5 ml (Fig. S11); this was not altered by the presence of MgATP, suggesting that MgATP binding is not inducing protein oligomerization under our assay conditions.

## Discussion

The mechanisms by which endogenous oxidants can modulate GS function is an important issue in many areas of human pathology and especially neurodegenerative diseases (19, 23, 24). Loss of GS function may have a profound impact on neurotransmitter metabolism *via* perturbation of glutamate-glutamine cycle, resulting in a decreased substrate availability for glutamatergic neurons and enhanced excitotoxicity due to decreased removal of extracellular glutamate (13). Data consistent with such effects has been reported in experimental animals subject to pharmacological GS inhibition (16, 28, 29). Here, we provide an in-depth biochemical and functional characterization of the oxidative modification of recombinant HsGS *in vitro*, to gain further information on a process that may be of biological relevance.

**Figure 12:**
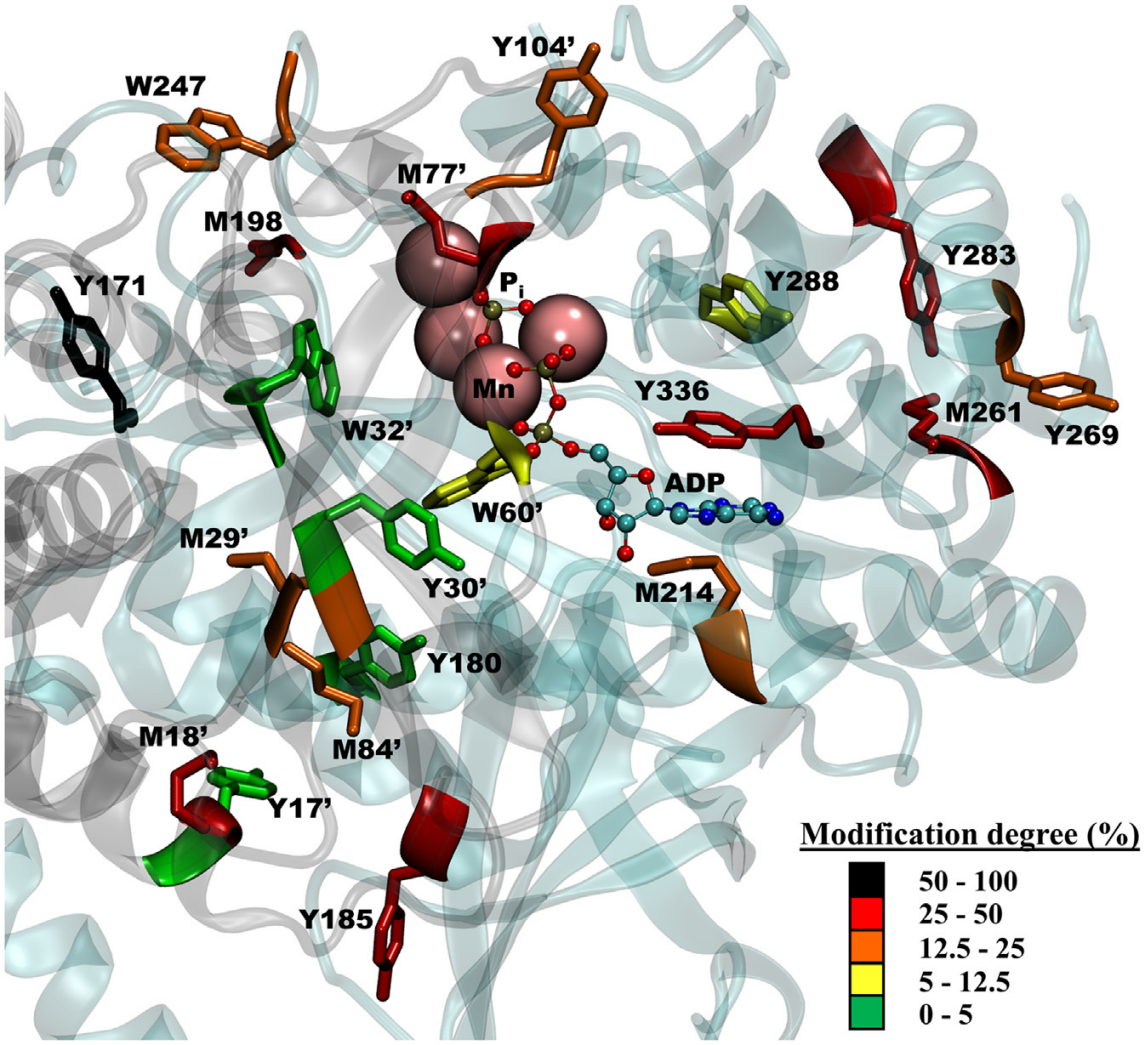
**Structural mapping of the main oxidatively modified residues by ONOO**^**−**^**in HsGS.** The peptide-mapping and relative quantification data obtained for ONOO^−^-treated HsGS was used to locate, in a subunit of an HsGS assembly, the Tyr, Met, and Trp residues that were found modified. The modification degree of each residue, as estimated through MS1 quantification of the Orbitrap Fusion analysis or through MRM analysis, is indicated by the color code of the specific amino acid. The 3D image of HsGS with Mn and ADP bound was rendered using VMD 1.9.3 from the PDB ID 2QC8 structure. MRM, Multiple Reaction Monitoring.

Through combined analytical, biochemical, and proteomic analyses, data has been obtained indicating that ONOO^−^ is capable of both causing a loss of enzyme activity (Fig. 1) and inducing HsGS aggregation (Fig. 7) through oxidative modification of a selective number of amino acids, namely, Cys, Tyr, Met, and Trp (Fig. 12). In contrast to what was previously reported (34), several Tyr residues were found to be nitrated by ONOO^−^ treatment (Fig. 2), being Tyr171, Tyr185, Tyr269, Tyr283, and Tyr336 the main targets‡.

**Figure 13:**
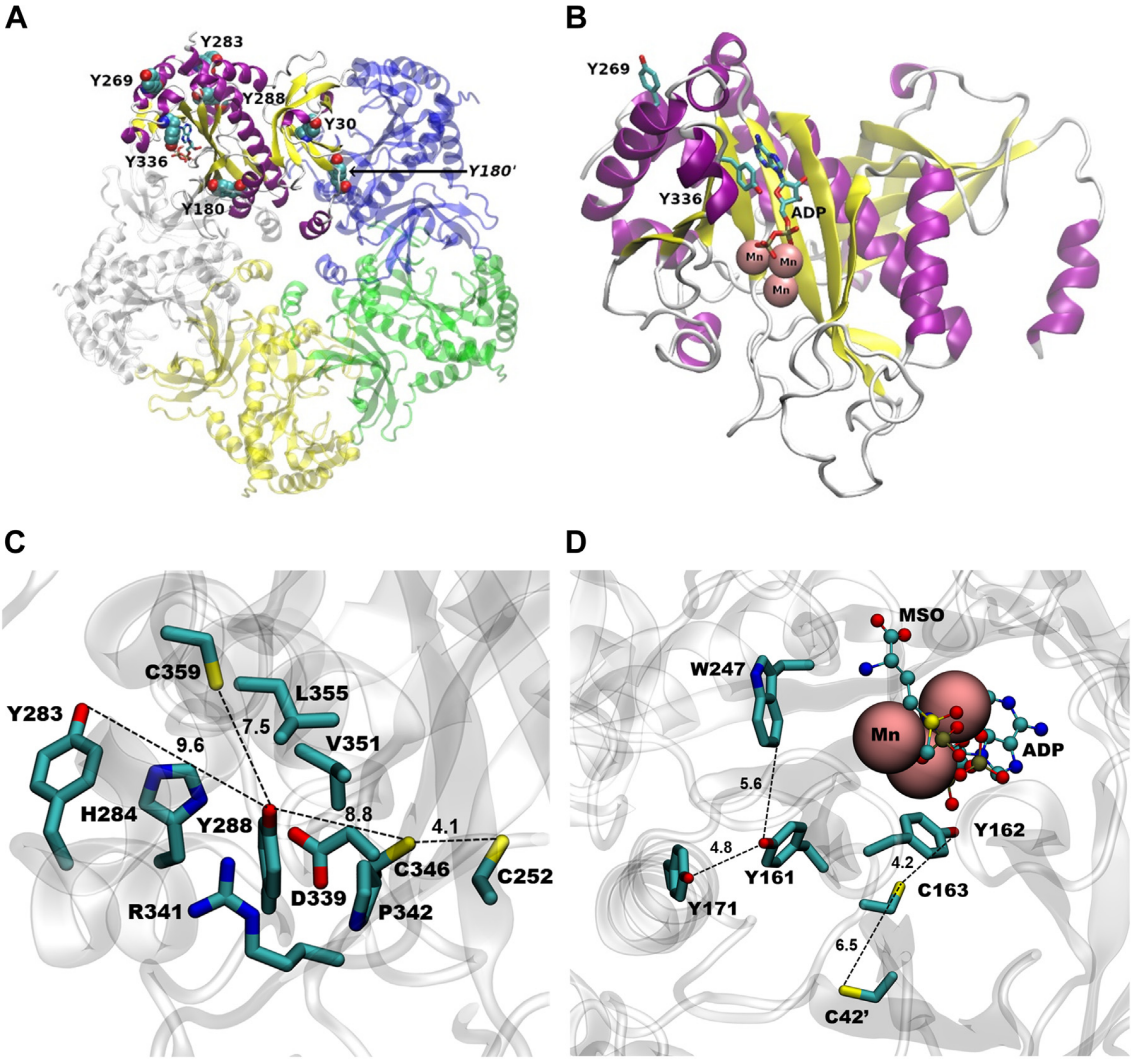
**Structural analysis of selected Tyr residues targeted by ONOO**^**−**^**-derived one-electron oxidants.***A*, overall structure of an HsGS pentameric ring, showing the location within a subunit of the six Tyr residues that were found to form DiTyr cross-links. *B*, specific location of the Tyr residues 269 and 336, both found to undergo nitration and dimerization, within a monomer. ADP and Mn^2+^ ions at the active site are also shown. *C*, detailed analysis of the structure surrounding Tyr288, including Tyr283 and Cys residues 252, 346, and 359. *D*, analysis of the main redox-active amino acids neighboring Tyr171 which could participate in electron-transfer reactions. ADP, L-methionine sulfoximine (MSO), and Mn^2+^ at the active site are also shown. In (*C*) and (*D*), the *dashed lines* indicates atom distances, which are expressed in Å. Structures were rendered in VMD VMD 1.9.3 from the PDB ID 2QC8 structure.

Along with nitration, Tyr dimerization to DiTyr was also found (Fig. 1). MS analysis revealed the formation of five different types of Tyr-Tyr cross-links that involved six different Tyr residues (Fig. S6), also found to be nitration targets. Through the structural analysis of the protein and considering the steric restrictions associated to DiTyr formation, it is possible to define if each cross-link is either intramolecular (between Tyr residues of a same monomer) or intermolecular (between Tyr residues of different monomers, either of the same or different decamers) (Fig. 13). For instance, the cross-links Tyr269-Tyr269 and Tyr336-Tyr336 are necessarily intermolecular between different decamers, since they are not close enough within a same decamer (Fig. 13, *A* and *B*); these two cross-links might participate in the aggregation of the protein. The Tyr283-Tyr288 cross-link is likely an intramolecular given the spatial distance between both side chains within a same monomer (Fig. 13*C*). The Tyr269-Tyr288 cross-link is rather difficult to explain (Fig. 13*A*); within a same polypeptide, the residues are too far away (˃20 Å) to assume the cross-link is intramolecular. Also, it is not likely that they are intermolecular inside a decamer because their distances within different subunits are even higher. However, given that Tyr288 is a buried residue, it is not easy to classify it as intermolecular between different decamers; the formation of this cross-link is possible secondary to previous amino acid modifications that alter the protein structure. Finally, the Tyr30-Tyr180 cross-link is likely an intermolecular union within a same decamer, given that both residues are too buried to interact with their counterparts in other decamers, but Tyr30 and Tyr180 from neighboring monomers of a pentameric ring locate at ∼10 Å (Fig. 13*A*). Some of the 3,3′-dityrosine cross-links may have an impact in protein structure and dynamics, the disclosure of which requires further work.

Given that the five HsGS Tyr→Phe mutants were susceptible to inactivation by ONOO^−^ (Fig. 6), the sole nitration at any of these targets is not responsible for enzyme inactivation. The Y171F mutant was inactivated by ONOO^−^ to a higher extent than the WT enzyme, suggesting that nitration of this residue, instead of being a relevant target for enzyme inactivation, acts more like a protective reaction that diverts oxidants away from the key targets. The high solvent exposure of Tyr171 might facilitate its oxidative modification; this could be supported by the proximity of several redox-active side chains that might stabilize Tyr171 radical (Fig. 13*D*). On the other hand, the initial oxidation of Tyr171 could be a way of oxidizing those neighboring residues through electron-transfer reactions (66); such process could eventually lead to either oxidation of active-site Tyr162 or to the formation of an intermolecular disulfide bonding between Cys163 and Cys42′. It is interesting to note that this finding contrasts previous results obtained for *Medicago truncatula* GS1a (32), for which it was proposed that nitration at Tyr167, sequence aligned with HsGS Tyr171, is responsible of the loss of enzyme activity. However, such conclusion was based only on the fact that the change of that residue to Phe made the protein much more resistant to inactivation by the synthetic nitrating agent tetranitromethane. Thus, the apparent differing findings may be due to multiple factors beyond the structural differences between the enzymes. Of particular interest is the Y288F mutant, because it had a very low specific activity compared to the WT; this suggests that alterations at this site, Tyr288, can have a strong impact on HsGS structure and function. Structural analysis reveals that Tyr288 is a buried residue with several charged side chains (His, Arg, and Asp residues) surrounding its -OH group (Fig. 13*C*); it is thus possible that either the loss of the -OH when the residue is changed to Phe or the alterations imposed by the incorporation of the -NO_2_ group can disrupt the interactions that are likely formed around this phenolic -OH and cause structural impairments. Given its low solvent exposure, the oxidative modification of this residue might be facilitated by the oxidation of Tyr283 (that is 9.6 Å away), which is highly solvent-exposed and was found to be a major nitration target. Thus, initial formation of a Tyr radical at this position may be a possible pathway for oxidizing Tyr288; this is further supported by the fact that a DiTyr cross-link was detected between Tyr283-Tyr288. Another interesting fact regarding Tyr288 is its proximity to two Cys residues, namely, Cys346 and Cys359 (Fig. 13*C*); it is possible therefore that a Tyr radical at Tyr288 might be repaired by either of them. In particular, if a Cys radical is formed in Cys346, the electron-transfer chain reactions might also include the neighboring Cys252 (∼4 Å) and, eventually, lead to a disulfide link between these two Cys residues.

The strongest previous hypothesis related to mammalian GS inactivation involves nitration of Tyr336, a conserved residue that interacts with adenine nucleotides in the active site (9). This hypothesis arose from *in vitro* experiments in which ovine GS was inactivated by ONOO^−^, with Tyr336, the only residue detected as nitrated by MS (34). This suggestion is supported by molecular dynamics simulations (38), in which an inactivation mechanism was proposed in which deprotonated (negatively-charged) NO_2_Tyr336, but not the neutral species, hampers ATP binding. This suggestion was supported by experiments carried out at pH 4.0 (at which NO_2_Tyr336 would be in its neutral state), which showed minimal inactivation. In the current study, Tyr336 was detected as a major site of nitration targets, supporting a role for this residue in the loss of HsGS function associated with ONOO^-^ exposure. However, our data suggests a more complex scenario in which Tyr336 nitration is only one of a series of oxidative events that lead to a loss of HsGS function. If nitration of Tyr336 was the sole determining factor responsible for HsGS inactivation by ONOO^−^, the Y336F mutant would be expected to be more resistant to inactivation than the WT enzyme, whereas the opposite was detected (Fig. 6*F*). This does not necessarily mean that Tyr336 nitration is not involved in the loss of HsGS activity, as the absence of Tyr336 might result in enhanced modification of other targets (Fig. 6*G*) that impact on activity. It is also important to note that there are significant experimental differences between the current work study and that of Görg *et al* (34) and Frieg *et al.* (38). These previous studies employed a GS concentration of 5 μg ml^−1^ (∼0.115 μM GS monomer), and ONOO^−^ excesses over the range 4 to 400:1, whereas here, the HsGS concentration was (for most experiments) 40 -fold higher (200 μg ml^−1^, ∼4.6 μM monomer), and ONOO^−^ excesses over the range ∼10 to 200:1. These differences may change the observed chemistry for two reasons: the ratio of direct *versus* indirect reactions of ONOO^−^ may vary, with two-electron oxidations favored under higher protein concentration conditions; and secondly, the oligomeric state of GS can be different, as the GS concentration is a determinant of this equilibrium (63). Other studies have reported modulating effects of high protein concentrations and crowding agents on protein aggregation (67, 68, 69). Furthermore, Frieg *et al.* measured GS activity through a coupled assay, which relies on the physiological GS reaction, unlike the one mostly used here. It is possible that this assay, which uses ATP, is more sensitive to inhibition than the γ-glutamyl transfer assay, which uses ADP in catalytic amounts. However, comparative studies carried out with both WT and Y336F HsGS performed here, under the same conditions used on previous reports, indicated that the Y336F mutant was even more readily inactivated than the WT (data not shown). So, these data do not support the hypothesis that Tyr336 nitration is the main cause of HsGS inactivation by ONOO^−^.

The observation that treatment of HsGS with ONOO^−^ results in aggregation and precipitation prompter examination of both early and late effects on the HsGS after addition of bolus ONOO^−^, as this may represent a biologically relevant process. Previous studies have reported similar effects under a range of conditions, including exposure to thiol alkylating agents, divalent metal ions, high concentrations, and freezing of the lyophilized protein (61, 62, 63, 64). Prokaryotic GS has also been reported to aggregate under certain conditions, with electron microscopy used to examine MnCl_2_-induced aggregation of *E. coli* GS (70). These observations imply that GS has a high susceptibility to aggregation, with oxidative damage also being a triggering-event. The results presented herein indicate that oxidation-induced aggregation of HsGS occurs due to oxidation of certain critical Cys residues. This finding suggest that some Cys residues are essential for the maintenance of protein structure and that their chemical modification, either by oxidation or alkylation (as reported previously (61, 62)), perturbs the native structure of HsGS and leads to its aggregation.

As shown in this work, HsGS aggregation induced by thiol oxidation is quantitatively relevant in terms of the overall loss of HsGS activity induced by oxidation. It represents a pathway to oxidant-associated loss of GS function under biological conditions that is not limited to ONOO^−^. Besides the *in vitro* data on GS aggregation, some *in vivo* evidence suggests that this may occur under certain conditions. Thus, in a mouse model of lateral amyotrophic sclerosis, GS was found in insoluble fractions of spinal cords at considerably higher levels than in control animals. Furthermore, the GS was also found to be nitrated, suggesting that oxidative stress associated with lateral amyotrophic sclerosis can promote both oxidative modification and aggregation of GS (40). Furthermore, in yeast cultures, it was observed that GS tends to form cytosolic foci and be enriched in insoluble cell lysate fractions under nutritional and thermal stress conditions (71). These events may arise *via* the thiol-mediated process outlined above. The mechanisms that connect Cys oxidation with aggregation still need to be defined, and specifically, identification of the Cys residues involved.

Another issue that remains to be addressed is how protein stability and quaternary structure influence both thiol oxidation and aggregation. Several studies have reported that ligand binding (especially adenine nucleotides and Mn^2+^/Mg^2+^) improve GS stability and prevent aggregation induced by several stimuli (61, 62, 65, 72). Thus, H_2_O_2_-induced thiol oxidation, aggregation, and loss of HsGS activity were prevented when oxidation was carried out in the presence of MgATP (Fig. 11, *A*–*D*). These changes were accompanied by an increased protein stability (Fig. 11*E*) but not by a change in the oligomeric state of the protein (Fig. S12). Therefore, further characterization on how ligand binding affects HsGS structure and stability is essential to understand the Cys oxidation and aggregation mechanisms of HsGS.

In conclusion, the data obtained herein indicates that the oxidative inactivation of HsGS by ONOO^−^ is a complex, multi-faceted process that occurs mainly through modification, including radical processes, of several amino acids, alterations to protein stability, and thiol oxidation–dependent aggregation of HsGS. The data provided herein helps to unravel biochemical mechanisms involved in HsGS inactivation and aggregation observed in aging and neurodegenerative diseases, revisiting the idea that a single oxidative modification such as tyrosine nitration accounts for the loss of function.

## Experimental procedures

### Reagents

All reagents were purchased from Merck unless otherwise specified. The plasmid pNIC28-Bsa4 containing the sequence corresponding to amino acids 5-365 of HsGS and a 22-amino acid N-terminal peptide containing a 6-histidine tag, as well as a TEV-protease recognition site (Construct ID GLULA-c004), was provided by the Structural Genomics Consortium (SGC) (9). Peroxynitrite was synthetized from sodium nitrite (NaNO_2_) and hydrogen peroxide (H_2_O_2_, Mallinckrodt Chemicals) in acid medium (73, 74). The ONOO^−^ stocks were kept in concentrated sodium hydroxide (NaOH) at −80 °C, with its concentration determined before use *via* its absorbance at 302 nm (ε = 1,670 M^−1^ cm^−1^) (75).

### HsGS expression

BL21 (DE3) star *E. coli* cells were transformed with the pNIC28-Bsa4 plasmid through the heat shock technique (76, 77). Transformed cells were selected by culturing the cells in LB plates supplemented with 50 μg ml^−1^ kanamycin (AppliChem). Then, precultures of LB medium (25 ml) containing 100 μg ml^−1^ kanamycin were inoculated with transformed colonies and incubated overnight at 37 °C with shaking in a MaxQ 6000 (Thermo Fisher Scientific) incubator. After this, cultures were transferred to a 1 l Terrific Broth medium containing 100 μg ml^−1^ kanamycin and incubated at 37 °C with shaking until they reached an optical density of 2 (∼4 h). HsGS expression was induced by addition of 0.5 mM IPTG (bioWORLD) with overnight incubation at 18 °C. Cells were then harvested (4,500*g*, 15 min) at 4 °C and pellets were resuspended in buffer A (50 mM sodium phosphate, pH 7.5 containing 0.5 M NaCl, 0.5 mM tris (2-carboxyethyl) phosphine hydrochloride, and 10% glycerol) supplemented with 10 mM imidazole and PMSF. The cell suspensions were then lysed by sonication (Branson Sonifier 450) and clarified by centrifugation (40 min, 15,000*g*, 4 °C). The supernatant was collected and used for HsGS purification.

### HsGS purification

The cell supernatant was filtered (0.2 μm cellulose acetate filter) and then passed through a 5 ml HisTrap HP (GE Healthcare) column loaded with nickel sulfate (NiSO_4_) previously equilibrated with buffer A containing 10 mM imidazole. The column was then washed successively with the following: buffer A solutions containing imidazole at 10 mM imidazole (50 ml); 25 mM imidazole (50 ml), 50 mM imidazole (40 ml), and 100 mM imidazole (25 ml). Aliquots were collected through the entire process (lysate loading and imidazole washes) to follow the purification process. Finally, recombinant HsGS was eluted by washing the column with buffer A containing 500 mM imidazole. Through the elution process, 1 ml aliquots were collected and protein content of each aliquot was assessed by its absorbance at 280 nm. Those with a 280 nm absorbance >0.5 were subjected to buffer exchange to buffer B (30 mM Hepes pH 7.5 containing 0.3 M NaCl, 2 mM tris (2-carboxyethyl) phosphine hydrochloride, and 10% glycerol) using a HiTrap desalting (GE Healthcare) column. Finally, HsGS samples in buffer B were concentrated using Amicon Ultra (Merck Millipore) centrifugal filters with a 100 kDa cutoff and stored at −80 °C in 200 μl aliquots (final concentration *∼*10 mg ml^−1^). This process yielded high purity HsGS with >15 mg protein l^−1^ of culture. The identity of the purified protein was confirmed by immunoblot analysis, activity assays, and protein mass spectrometry (Fig. S1).

### Activity assays

GS activity was assessed through a γ-glutamyl transferase reaction assay, based on the GS-catalyzed synthesis of γ-glutamyl hydroxamate (GlnNHOH) from glutamine and hydroxylamine (NH_2_OH) in the presence of Mn^2+^, sodium arsenate (Na_2_HAsO_4_), and ADP, as described previously (34, 78). Typically, the assay was performed as follows: HsGS (10 nM decamer concentration) was incubated for 15 min at 37 °C with 60 mM L-glutamine, 30 mM NH_2_OH, 20 mM Na_2_HAsO_4_, 0.4 mM ADP, and 1.5 mM MnCl_2_ in 60 mM imidazole buffer pH 6.8 (total final volume of 250 μl). Reaction was stopped by the addition of an equal volume of stop solution (0.37 M FeCl_3_, 0.2 M trichloroacetic acid in 0.67 HCl). The product yield (GlnNHOH) was measured spectrophotometrically at 500 nm (corresponding to a [Fe^3+^-GlnNHOH] complex) in a 96-well plate reader (Varioskan Flash, Thermo Fisher Scientific) and its concentration calculated *via* a calibration curve constructed using authentic GlnNHOH. The assay conditions described were established after performing temporal courses of GlnNHOH synthesis, in order to guarantee initial rate conditions under the conditions employed. GS activity was also measured through inorganic phosphate quantification produced as a product of the physiological reaction catalyzed by GS (glutamine synthesis from L-glutamate, ammonia, and ATP) as reported previously (79), as well as by using a variation of this reaction, in which NH_3_ was replaced by NH_2_OH, leading to the ATP-dependent synthesis of GlnNHOH which was quantified at 500 nm (61).

### HsGS exposure to ONOO^−^ and other oxidants

Before reactions were carried out, HsGS aliquots in buffer B were incubated with 10 mM DTT for 30 min on ice and then desalted using 100 mM potassium phosphate (KPi) buffer pH 7.3 containing 0.1 M KCl and 0.1 mM DTPA. After this buffer exchange, the protein concentration was determined using the bicinchoninic acid assay (Sigma) (80). Exposure of HsGS to ONOO^−^ was typically carried out in 200 μl samples containing 0.2 mg ml^−1^ HsGS (4.62 μM monomer) in 100 mM KPi pH 7.3 containing 0.1 M KCl and 0.1 mM DTPA. Different ONOO^−^ concentrations were added in a volume of 5 μl at 21 °C and after 5 min incubation, aliquots were taken for analysis. The pH of the samples was determined after ONOO^−^ addition to verify minimal changes (<0.1 pH units). As a matching control condition, reverse order addition of ONOO^−^ was also performed by allowing ONOO^−^ to decompose in KPi buffer before HsGS was added to the reaction samples. The reverse order addition experiment was aimed to confirm the effects due to ONOO^−^ itself and not to decay products (mostly NO_3_^−^) or remaining amounts of precursors from the synthesis of ONOO^−^ (*i.e.*, NO_2_^−^ and H_2_O_2_). In some experiments, 1 mM DF was added prior to ONOO^−^ addition. To study the effect of CO_2_, reactions were carried out as described above, but in 100 mM KPi buffer pH 7.3 containing 0.1 M KCl, 0.1 mM DTPA, and 25 mM NaHCO_3_. To study the pH dependency of the reactions, the HsGS was initially desalted to 0.1 M KCl and then exposed to 0.5 mM ONOO^−^ in 100 mM KPi buffer at pH values ranging from 6.1 to 8.3, containing 0.1 M KCl and 0.1 mM DTPA.

Oxidation of HsGS by H_2_O_2_ was carried out by incubating 0.2 mg protein ml^−1^ with varying concentrations of H_2_O_2_ in 100 mM KPi buffer pH 7.3 containing 0.1 M KCl and 0.1 mM DTPA for 1 h at 37 °C; reactions were stopped by addition of 10 nM catalase (Fluka BioChemika). Oxidation by peroxyl radicals used the thermolabile donor ABAP (Wako) with HsGS incubated under similar conditions with 10 mM ABAP at 37 °C for 120 min (0.3 μM min^−1^ peroxyl radicals (81)) with aliquots removed at different times for analysis. Diamide oxidation was performed by exposing HsGS to different concentrations of diamide in 100 mM KPi buffer pH 7.3 containing 0.1 M KCl and 0.1 mM DTPA at 37 °C for 1 h.

### SDS-PAGE, immunoblot, and dot blot analysis

Electrophoretic characterization of HsGS samples exposed to ONOO^−^ and other oxidants was carried out using 10% SDS-PAGE gels in the absence or presence of β-mercaptoethanol. For immunoblot analysis, SDS-PAGE–separated samples were transferred to a nitrocellulose membrane (Odyssey, Li-Cor Biosciences) using a semi-dry electrotransfer Hoefer TE 70 device (Amersham Biosciences). The membrane was then blocked with 5% milk in PBS for 1 h at 21 °C, washed with PBS containing 0.1% Tween, and then successively incubated with either monoclonal anti-NO_2_Tyr antibody (Invitrogen, clone number HM11, 1/1000) or monoclonal anti-DiTyr antibody (JaICA, clone number 1C3, 1/1000) and polyclonal anti-GS antibody (Abcam, 1/2000). After washing, secondary antibodies conjugated with IR800 and IR680 dyes (Li-Cor Biosciences) were added and the membrane was finally imaged using a Li-Cor Odyssey fluorescence imager. Dot blot analysis was carried out analogously after direct addition of 2.5 μl of samples to a nitrocellulose membrane; after developing the image, signals were quantified by densitometry, and NO_2_Tyr and DiTyr intensities were normalized to their corresponding GS signal (NO_2_Tyr/GS or DiTyr/GS intensity ratios) and expressed relative to untreated controls. All image acquisition and processing was done using the Image Studio (Li-Cor) software (https://www.licor.com/bio/image-studio/).

### Spectrophotometric quantification of protein thiols and NO_2_Tyr

The thiol content of HsGS was determined spectrophotometrically by reaction with 5,5′-dithiobis(2-nitrobenzoic acid). Oxidant-exposed HsGS was precipitated by the addition of ice-cold acetone and incubating for 20 min at −20 °C. After centrifugation (18,000*g*, 30 min, 4 °C), the pellets were dissolved in 6 M guanidine chloride prepared in 20 mM Tris pH 7.2, and 1 mM 5,5′-dithiobis(2-nitrobenzoic acid) was added from a 20 mM stock prepared in ethanol. After 30 min incubation at 21 °C (in the dark), thiol levels were quantified spectrophotometrically at 412 nm (ε = 13,880 M^−1^ cm^−1^) (82). Absolute NO_2_Tyr concentrations were measured for some conditions using a similar procedure. Briefly, after HsGS exposure to ONOO^−^, the protein was precipitated and redissolved (as above) in a volume that was 20% of the original volume (*i.e.*, 5-fold concentration). After this, pH of samples was adjusted to 10 using NaOH, and the NO_2_Tyr content quantified at 430 nm (ε = 4,100 M^−1^ cm^−1^) (83).

### UHPLC quantification of HsGS amino acids

The amino acid content of native and ONOO^−^-treated HsGS was performed using acid hydrolysis with methanesulfonic acid followed by UHPLC separation and quantification of tagged amino acids as reported previously (84, 85). Briefly, HsGS samples (200 μg, in glass hydrolysis vials) were precipitated with 10% trichloroacetic acid, pellets were washed twice with ice-cold acetone, and resuspended in 4 M methanesulfonic acid containing 0.2% (w/v) tryptamine. Oxygen was then removed and samples were incubated overnight under vacuum at 110 °C using a Pico-Tag system. The samples, once cool, were neutralized with NaOH, filtered through 0.2 μm Pall Nanosep filters, and diluted 10- and 100-fold with H_2_O. Amino acid analysis was finally carried out on a Shimadzu Nexera UHPLC system with precolumn derivatization of the amino acids using the fluorescent tag *o*-phthaldialdehyde, with the amino acids separated on a reverse phase column (Phenomenex Kinetex 2.6 μm EVO 150 × 3 mm, 40 °C), with detection of the labeled amino acids monitored using λ_ex_ 340 nm and λ_em_ 440 nm (85). A commercial amino acid mixture (Sigma) was used for peak identification and quantification. The concentrations of each amino acid were corrected for the dilution steps to give the concentrations present in the reaction tubes.

### Identification of nitro-oxidative modifications in HsGS by mass spectrometry

Different mass spectrometry approaches were used to identify residues modified by ONOO^−^ exposure. Initial identification of NO_2_Tyr-modified residues was done using a QTRAP 4500 (ABSciex) triple quadrupole mass spectrometer coupled to a HPLC system (Agilent) using an approach based on the detection of the NO_2_Tyr-derived immonium ion (*m/z* 181) and fragmentation of the precursor ions as described previously (52, 54). For this analysis, 40 μg of ONOO^−^-treated HsGS was digested with trypsin (Sigma) overnight at 37 °C according to standard in-solution digestion protocols (86). The resulting peptide mixture was lyophilized, dissolved in nanopure water to a concentration corresponding to 9.24 μM protein monomer, and subjected to HPLC-MS/MS analysis. Data was analyzed using the Mascot web server (Matrix Science) for identifying the peptides containing NO_2_Tyr residues (Table S1).

A deeper MS/MS analysis was performed following a full MS Orbitrap scan and data-dependent tandem mass spectrometry according to the procedure described in ref (55), which included trypsin digestion of ONOO^−^-treated samples in both H_2_^16^O and H_2_^18^O (55, 85). Briefly, a total amount of 20 μg of HsGS was digested (half in H_2_^16^O and half in H_2_^18^O containing buffers); peptides obtained from both digests were then subjected to solid-phase extraction using StageTip C18 reversed-phase discs packed into pipette tips as previously reported (55, 87, 88), dried in a Speedvac concentrator after elution, and finally resuspended in 10 μl of H_2_^16^O and H_2_^18^O, respectively. The digested peptides were mixed 1:1 and immediately separated and analyzed on an EASY-nLC 1000 chromatograph coupled to an Orbitrap Fusion mass spectrometer (Thermo Fisher Scientific). The raw data was analyzed using MaxQuant 1.6.1.0 (https://www.maxquant.org/) (89) for the identification of non–cross-linked peptides in their native and modified forms (Table S2). To determine the extent of formation of each of the identified oxidative modifications, the results obtained from the MaxQuant analysis, along with the raw data, were reanalyzed using Skyline 4.1.0 software (https://skyline.ms/project/home/begin.view) (53). This allowed for the quantification of the MS1 features corresponding to each of the identified peptides and from the area of the peaks, the extent (percentage) modification at each site in the peptides. The identification of protein cross-links was done as described in ref (55), using the MassAI software (University of Southern Denmark; https://www.massai.dk/). Identified cross-links were validated by detection of a + 8 Da mass shift in cross-linked peptides due to ^18^O isotopic labeling during trypsin digestion in H_2_^18^O.

The MS/MS fragmentation data obtained by Orbitrap analysis and MaxQuant peptide identification was used to elaborate an MRM-targeted method to specifically detect the main native and modified peptides (Table S3). This permitted an estimate to be made of the percentage modification of each peptide, under varying conditions, from the QTRAP 4500 triple quadrupole MS. Each peptide was detected by simultaneously following three different transitions (Q1: parent ion; Q3: three selected *y* or *b* ions, which gave intense signals and, preferentially, contained the residues that undergo oxidation); the peak areas of the native peptides and its modified variants were obtained using the Analyst Software (Sciex, https://sciex.com/products/software/analyst-software) and used for estimating the extent of modification at each residue.

### HsGS aggregation

Oxidant-induced aggregation of HsGS was followed by turbidity measurements over time as described previously (90). HsGS samples (0.2 mg ml^−1^) were exposed to the different oxidants at 21 °C as described above and then transferred to a 96-well plate. After this, the samples were covered with 80 μl of mineral oil and the absorbance at 405 nm measured every 5 min for 12 h at 37 °C using a Varioskan Flash microplate reader (Thermo Fisher Scientific). To study if aggregated HsGS was able to induce the aggregation of native protein, the following procedure was carried out: HsGS was treated with 500 μM ONOO^−^, allowed to aggregate at 37 °C for 90 min, and then different amounts of the aggregated material (0, 5, 10, and 20 μg) were mixed with native HsGS (0.2 mg ml^−1^); aggregation of the mixtures was subsequently studied as described above.

### Separation of insoluble and soluble fractions of oxidized HsGS

Separation of insoluble aggregated HsGS from soluble protein was performed after exposure of HsGS (0.5 mg ml^−1^) to 500 μM ONOO^−^ in KPi buffer at 21 °C. The samples were then incubated at 37 °C for 30 min to allow aggregation to occur. After this, the samples were centrifuged for 30 min at 18,500*g* (4 °C) to remove insoluble material. Finally, the supernatants were collected and the remaining protein quantified by the bicinchoninic acid assay. Specific HsGS activity was measured in the supernatants (30 min, soluble fraction) and compared to that of mixtures immediately (0 min) and 30 min after ONOO^−^ exposure. For MS analysis, pellets were dissolved in 8 M urea prepared in 50 mM tris buffer pH 8.0 and subjected to trypsin digestion as described above; the supernatants were first vacuum-dried and then subjected to an identical procedure.

### Thermal denaturation studies

The stability of HsGS was studied by following tryptophan fluorescence (λ_ex_ 295 nm, λ_ex_ 335 nm) in a Jasco Spectrofluorimeter. Briefly, HsGS (0.2 mg ml^−1^) was incubated either without or with 20 mM MgCl_2_, 20 mM MgCl_2_, and 10 mM ATP, or after exposure to 50 μM ONOO^−^ in a final volume of 500 μl, and fluorescence was recorded at temperatures ranging from 15 to 65 °C (with a temperature ramp of 1.5 °C min^−1^). The data obtained were fitted to a thermal denaturation curve considering nonlinear baselines (91) to obtain the apparent melting temperature (Tm) values.

### Gel filtration studies

The oligomeric state of HsGS and the influence of MgATP binding on it was studied on a Superdex 200 10/300 Gl (GE Healthcare) gel filtration column. As mobile phase, a 20 mM phosphate buffer pH 7.4 containing 100 mM KCl, 0.1 mM DTPA, and 1 mM β-mercaptoethanol was used; when studying the effect of MgATP binding, the buffer was supplemented with 5 mM ATP and 20 mM MgCl_2_. For each run, 100 μl of 4 mg ml^−1^ HsGS prepared in the mobile phase buffer (with or without MgATP) was injected and eluted at a flow rate of 0.4 ml min^−1^ over 75 min with continuous measurement of absorbance at 280 nm. Molecular weight calibration of the column was done using a commercial gel filtration markers kit (Merck) in the range 12 to 200 kDa, with the inclusion also of ferritin (MW 440 kDa); blue dextran was used for void volume determination.

### Statistical analysis

Results are expressed as mean ± SD of at least three independent experiments. Statistical analysis was performed using GraphPad Prism 6 software (https://www.graphpad.com/scientific-software/prism/). Comparisons between two groups were carried out using an unpaired t test analysis, while comparisons between more than two data sets were performed through one-way ANOVA analysis with Tukey *post hoc* testing; in both cases, *p* ˂ 0.05 was considered statistically significant.

## Data availability

All data described are contained within the article. The Orbitrap Fusion peptide mapping-mass spectrometry data have been deposited to the ProteomeXchange Consortium *via* the PRIDE (92) partner repository with the dataset identifier PXD037583. Additional data are available upon request.

## Supporting information

This article contains supporting information.

## Conflict of interest

The authors declare that they have no conflicts of interest with the content of this article.

## Footnotes

† IUPAC-recommended names for peroxynitrite anion (ONOO^−^) and peroxynitrous acid (ONOOH) (p*Ka* = 6.8) are oxoperoxonitrate (1-) and hydrogen oxoperoxonitrate, respectively. The term peroxynitrite is used (unless specified) to refer to the sum of ONOO^−^ and ONOOH.

‡ Tyr104 was left off the list of nitrated residues of significance because a basal level of nitration of it was detected in the control, which was increased in the ROA condition.

## References

[1] van Rooyen J.M., Abratt V.R., Belrhali H., Sewell T. (2011). Crystal structure of type III glutamine synthetase: surprising reversal of the inter-ring interface. Structure.

[2] Pesole G., Bozzetti M.P., Lanave C., Preparata G., Saccone C. (1991). Glutamine synthetase gene evolution: a good molecular clock. Proc. Natl. Acad. Sci. U. S. A..

[3] Kumada Y., Benson D.R., Hillemann D., Hosted T.J., Rochefort D.A., Thompson C.J. (1993). Evolution of the glutamine synthetase gene, one of the oldest existing and functioning genes. Proc. Natl. Acad. Sci. U. S. A..

[4] Bernard S.M., Habash D.Z. (2009). The importance of cytosolic glutamine synthetase in nitrogen assimilation and recycling. New Phytol..

[5] Mathis R., Gamas P., Meyer Y., Cullimore J.V. (2000). The presence of GSI-like genes in higher plants: support for the paralogous evolution of GSI and GSII genes. J. Mol. Evol..

[6] Wistow G., Bernstein S.L., Wyatt M.K., Behal A., Touchman J.W., Bouffard G. (2002). Expressed sequence tag analysis of adult human lens for the NEIBank project: over 2000 non-redundant transcripts, novel genes and splice variants. Mol. Vis..

[7] Wyatt K., White H.E., Wang L., Bateman O.A., Slingsby C., Orlova E.V. (2006). Lengsin is a survivor of an ancient family of class I glutamine synthetases re-engineered by evolution for a role in the vertebrate lens. Structure.

[8] Unno H., Uchida T., Sugawara H., Kurisu G., Sugiyama T., Yamaya T. (2006). Atomic structure of plant glutamine synthetase: a key enzyme for plant productivity. J. Biol. Chem..

[9] Krajewski W.W., Collins R., Holmberg-Schiavone L., Jones T.A., Karlberg T., Mowbray S.L. (2008). Crystal structures of mammalian glutamine synthetases illustrate substrate-induced conformational changes and provide opportunities for drug and herbicide design. J. Mol. Biol..

[10] Yamashita M.M., Almassy R.J., Janson C.A., Cascio D., Eisenberg D. (1989). Refined atomic model of glutamine synthetase at 3.5 A resolution. J. Biol. Chem..

[11] Eisenberg D., Gill H.S., Pfluegl G.M., Rotstein S.H. (2000). Structure-function relationships of glutamine synthetases. Biochim. Biophys. Acta.

[12] Matthews G.D., Gould R.M., Vardimon L. (2005). A single glutamine synthetase gene produces tissue-specific subcellular localization by alternative splicing. FEBS Lett..

[13] Popoli M., Yan Z., McEwen B.S., Sanacora G. (2011). The stressed synapse: the impact of stress and glucocorticoids on glutamate transmission. Nat. Rev. Neurosci..

[14] Qvartskhava N., Lang P.A., Gorg B., Pozdeev V.I., Ortiz M.P., Lang K.S. (2015). Hyperammonemia in gene-targeted mice lacking functional hepatic glutamine synthetase. Proc. Natl. Acad. Sci. U. S. A..

[15] Suarez I., Bodega G., Fernandez B. (2002). Glutamine synthetase in brain: effect of ammonia. Neurochem. Int..

[16] Robinson S.R. (2000). Neuronal expression of glutamine synthetase in Alzheimer's disease indicates a profound impairment of metabolic interactions with astrocytes. Neurochem. Int..

[17] Kulijewicz-Nawrot M., Sykova E., Chvatal A., Verkhratsky A., Rodriguez J.J. (2013). Astrocytes and glutamate homoeostasis in Alzheimer's disease: a decrease in glutamine synthetase, but not in glutamate transporter-1, in the prefrontal cortex. ASN Neuro.

[18] Robinson S.R. (2001). Changes in the cellular distribution of glutamine synthetase in Alzheimer's disease. J. Neurosci. Res..

[19] Le Prince G., Delaere P., Fages C., Lefrancois T., Touret M., Salanon M. (1995). Glutamine synthetase (GS) expression is reduced in senile dementia of the Alzheimer type. Neurochem. Res..

[20] Li K.Y., Gong P.F., Li J.T., Xu N.J., Qin S. (2020). Morphological and molecular alterations of reactive astrocytes without proliferation in cerebral cortex of an APP/PS1 transgenic mouse model and Alzheimer's patients. Glia.

[21] Olabarria M., Noristani H.N., Verkhratsky A., Rodriguez J.J. (2011). Age-dependent decrease in glutamine synthetase expression in the hippocampal astroglia of the triple transgenic Alzheimer's disease mouse model: mechanism for deficient glutamatergic transmission?. Mol. Neurodegener.

[22] Souza D.G., Bellaver B., Raupp G.S., Souza D.O., Quincozes-Santos A. (2015). Astrocytes from adult Wistar rats aged *in vitro* show changes in glial functions. Neurochem. Int..

[23] Hensley K., Hall N., Subramaniam R., Cole P., Harris M., Aksenov M. (1995). Brain regional correspondence between Alzheimer's disease histopathology and biomarkers of protein oxidation. J. Neurochem..

[24] Smith C.D., Carney J.M., Starke-Reed P.E., Oliver C.N., Stadtman E.R., Floyd R.A. (1991). Excess brain protein oxidation and enzyme dysfunction in normal aging and in Alzheimer disease. Proc. Natl. Acad. Sci. U. S. A..

[25] Butterfield D.A., Poon H.F., St Clair D., Keller J.N., Pierce W.M., Klein J.B. (2006). Redox proteomics identification of oxidatively modified hippocampal proteins in mild cognitive impairment: insights into the development of Alzheimer's disease. Neurobiol. Dis..

[26] Castegna A., Aksenov M., Aksenova M., Thongboonkerd V., Klein J.B., Pierce W.M. (2002). Proteomic identification of oxidatively modified proteins in Alzheimer's disease brain. Part I: creatine kinase BB, glutamine synthase, and ubiquitin carboxy-terminal hydrolase L-1. Free Radic. Biol. Med..

[27] Shen L., Chen C., Yang A., Chen Y., Liu Q., Ni J. (2015). Redox proteomics identification of specifically carbonylated proteins in the hippocampi of triple transgenic Alzheimer's disease mice at its earliest pathological stage. J. Proteomics.

[28] Ng K.T., O'Dowd B.S., Rickard N.S., Robinson S.R., Gibbs M.E., Rainey C. (1997). Complex roles of glutamate in the Gibbs-Ng model of one-trial aversive learning in the new-born chick. Neurosci. Biobehav Rev..

[29] Gibbs M.E., O'Dowd B.S., Hertz L., Robinson S.R., Sedman G.L., Ng K.T. (1996). Inhibition of glutamine synthetase activity prevents memory consolidation. Brain Res. Cogn. Brain Res..

[30] Berlett B.S., Friguet B., Yim M.B., Chock P.B., Stadtman E.R. (1996). Peroxynitrite-mediated nitration of tyrosine residues in *Escherichia coli* glutamine synthetase mimics adenylylation: Relevance to signal transduction. Proc. Natl. Acad. Sci. U. S. A..

[31] Berlett B.S., Levine R.L., Stadtman E.R. (1998). Carbon dioxide stimulates peroxynitrite-mediated nitration of tyrosine residues and inhibits oxidation of methionine residues of glutamine synthetase: both modifications mimic effects of adenylylation. Proc. Natl. Acad. Sci. U. S. A..

[32] Melo P.M., Silva L.S., Ribeiro I., Seabra A.R., Carvalho H.G. (2011). Glutamine synthetase is a molecular target of nitric oxide in root nodules of *Medicago truncatula* and is regulated by tyrosine nitration. Plant Physiol..

[33] Görg B., Qvartskhava N., Voss P., Grune T., Häussinger D., Schliess F. (2007). Reversible inhibition of mammalian glutamine synthetase by tyrosine nitration. FEBS Lett..

[34] Gorg B., Wettstein M., Metzger S., Schliess F., Haussinger D. (2005). Lipopolysaccharide-induced tyrosine nitration and inactivation of hepatic glutamine synthetase in the rat. Hepatology.

[35] Gorg B., Bidmon H.J., Keitel V., Foster N., Goerlich R., Schliess F. (2006). Inflammatory cytokines induce protein tyrosine nitration in rat astrocytes. Arch. Biochem. Biophys..

[36] Blough N.V., Zafiriou O.C. (1985). Reaction of superoxide with nitric oxide to form peroxonitrite in alkaline aqueous solution. Inorg. Chem..

[37] Radi R. (2004). Nitric oxide, oxidants, and protein tyrosine nitration. Proc. Natl. Acad. Sci. U. S. A..

[38] Frieg B., Gorg B., Qvartskhava N., Jeitner T., Homeyer N., Haussinger D. (2020). Mechanism of fully reversible, pH-sensitive inhibition of human glutamine synthetase by tyrosine nitration. J. Chem. Theor. Comput..

[39] Schliess F., Gorg B., Fischer R., Desjardins P., Bidmon H.J., Herrmann A. (2002). Ammonia induces MK-801-sensitive nitration and phosphorylation of protein tyrosine residues in rat astrocytes. FASEB J..

[40] Basso M., Samengo G., Nardo G., Massignan T., D'Alessandro G., Tartari S. (2009). Characterization of detergent-insoluble proteins in ALS indicates a causal link between nitrative stress and aggregation in pathogenesis. PLoS One.

[41] Peinado M.Á., Hernández R., Peragón J., Ovelleiro D., Pedrosa J.Á., Blanco S. (2014). Proteomic characterization of nitrated cell targets after hypobaric hypoxia and reoxygenation in rat brain. J. Proteomics.

[42] Alvarez B., Ferrer-Sueta G., Freeman B.A., Radi R. (1999). Kinetics of peroxynitrite reaction with amino acids and human serum albumin. J. Biol. Chem..

[43] Ferrer-Sueta G., Campolo N., Trujillo M., Bartesaghi S., Carballal S., Romero N. (2018). Biochemistry of peroxynitrite and protein tyrosine nitration. Chem. Rev..

[44] Davies M.J. (2016). Protein oxidation and peroxidation. Biochem. J..

[45] Radi R. (2013). Peroxynitrite, a stealthy biological oxidant. J. Biol. Chem..

[46] Fernandes S.P., Dringen R., Lawen A., Robinson S.R. (2011). Inactivation of astrocytic glutamine synthetase by hydrogen peroxide requires iron. Neurosci. Lett..

[47] Kimura K., Sugano S. (1992). Inactivation of *Bacillus subtilis* glutamine synthetase by metal-catalyzed oxidation. J. Biochem..

[48] Levine R.L. (1983). Oxidative modification of glutamine synthetase. II. Characterization of the ascorbate model system. J. Biol. Chem..

[49] Aksenov M.Y., Aksenova M.V., Carney J.M., Butterfield D.A. (1997). Oxidative modification of glutamine synthetase by amyloid beta peptide. Free Radic. Res..

[50] Souza J.M., Peluffo G., Radi R. (2008). Protein tyrosine nitration--functional alteration or just a biomarker?. Free Radic. Biol. Med..

[51] Campolo N., Issoglio F.M., Estrin D.A., Bartesaghi S., Radi R. (2020). 3-Nitrotyrosine and related derivatives in proteins: precursors, radical intermediates and impact in function. Essays Biochem..

[52] Batthyany C., Bartesaghi S., Mastrogiovanni M., Lima A., Demicheli V., Radi R. (2017). Tyrosine-nitrated proteins: proteomic and bioanalytical aspects. Antioxid. Redox Signal.

[53] Schilling B., Rardin M.J., MacLean B.X., Zawadzka A.M., Frewen B.E., Cusack M.P. (2012). Platform-independent and label-free quantitation of proteomic data using MS1 extracted ion chromatograms in skyline: application to protein acetylation and phosphorylation. Mol. Cell Proteomics.

[54] Petersson A.S., Steen H., Kalume D.E., Caidahl K., Roepstorff P. (2001). Investigation of tyrosine nitration in proteins by mass spectrometry. J. Mass Spectrom..

[55] Mariotti M., Leinisch F., Leeming D.J., Svensson B., Davies M.J., Hagglund P. (2018). Mass-spectrometry-based identification of cross-links in proteins exposed to photo-oxidation and peroxyl radicals using ^18^O labeling and optimized tandem mass spectrometry fragmentation. J. Proteome Res..

[56] Hagglund P., Mariotti M., Davies M.J. (2018). Identification and characterization of protein cross-links induced by oxidative reactions. Expert Rev. Proteomics.

[57] Denicola A., Souza J.M., Gatti R.M., Augusto O., Radi R. (1995). Desferrioxamine inhibition of the hydroxyl radical-like reactivity of peroxynitrite: role of the hydroxamic groups. Free Radic. Biol. Med..

[58] Bartesaghi S., Trujillo M., Denicola A., Folkes L., Wardman P., Radi R. (2004). Reactions of desferrioxamine with peroxynitrite-derived carbonate and nitrogen dioxide radicals. Free Radic. Biol. Med..

[59] Davies M.J., Donkor R., Dunster C.A., Gee C.A., Jonas S., Willson R.L. (1987). Desferrioxamine (Desferal) and superoxide free radicals. Formation of an enzyme-damaging nitroxide. Biochem. J..

[60] Mariotti M., Rogowska-Wrzesinska A., Hagglund P., Davies M.J. (2021). Cross-linking and modification of fibronectin by peroxynitrous acid: mapping and quantification of damage provides a new model for domain interactions. J. Biol. Chem..

[61] Ronzio R.A., Wilk S., Rowe W.B., Meister A. (1969). Preparation and studies on the characterization of sheep brain glutamine synthetase. Biochemistry.

[62] Wilk S., Meister A., Haschemeyer R.H. (1969). Studies on the subunit structure of ovine brain glutamine synthetase. Biochemistry.

[63] Denman R.B., Wedler F.C. (1984). Association-dissociation of mammalian brain glutamine synthetase: effects of metal ions and other ligands. Arch. Biochem. Biophys..

[64] Wilk S., Meister A., Haschemeyer R.H. (1970). Dissociation of native octameric brain glutamine synthetase to a tetramer by treatment with N-acetylimidazole. Biochemistry.

[65] Rao D.R., Beyreuther K., Jaenicke L. (1973). A comparative study of pig and sheep-brain glutamine synthetases: tryptic peptides and thiol groups. Eur. J. Biochem..

[66] Martinez A., Peluffo G., Petruk A.A., Hugo M., Piñeyro D., Demicheli V. (2014). Structural and molecular basis of the peroxynitrite-mediated nitration and inactivation of *Trypanosoma cruzi* iron-superoxide dismutases (Fe-SODs) A and B: disparate susceptibilities due to the repair of Tyr35 radical by Cys83 in Fe-SODB through intramolec. J. Biol. Chem..

[67] Aicardo A., Mastrogiovanni M., Cassina A., Radi R. (2018). Propagation of free-radical reactions in concentrated protein solutions. Free Radic. Res..

[68] Fuentes-Lemus E., Reyes J.S., Gamon L.F., Lopez-Alarcon C., Davies M.J. (2021). Effect of macromolecular crowding on protein oxidation: consequences on the rate, extent and oxidation pathways. Redox Biol..

[69] Biswas S., Bhadra A., Lakhera S., Soni M., Panuganti V., Jain S. (2021). Molecular crowding accelerates aggregation of alpha-synuclein by altering its folding pathway. Eur. Biophys. J..

[70] Valentine R.C., Shapiro B.M., Stadtman E.R. (1968). Regulation of glutamine synthetase. XII. Electron microscopy of the enzyme from *Escherichia coli*. Biochemistry.

[71] O'Connell J.D., Tsechansky M., Royal A., Boutz D.R., Ellington A.D., Marcotte E.M. (2014). A proteomic survey of widespread protein aggregation in yeast. Mol. Biosyst..

[72] Pamiljans V., Krishnaswamy P.R., Dumville G., Meister A. (1962). Studies on the mechanism of glutamine synthesis; isolation and properties of the enzyme from sheep brain. Biochemistry.

[73] Saha A., Goldstein S., Cabelli D., Czapski G. (1998). Determination of optimal conditions for synthesis of peroxynitrite by mixing acidified hydrogen peroxide with nitrite. Free Radic. Biol. Med..

[74] Reed J.W., Ho H.H., Jolly W.L. (1974). Chemical syntheses with a quenched flow reactor. Hydroxytrihydroborate and peroxynitrite. J. Am. Chem. Soc..

[75] Hughes M.N., Nicklin H.G. (1968). The chemistry of pernitrites. Part I. Kinetics of decomposition of pernitrous acid. J. Chem. Soc. A..

[76] Mandel M., Higa A. (1970). Calcium-dependent bacteriophage DNA infection. J. Mol. Biol..

[77] Hanahan D. (1983). Studies on transformation of *Escherichia coli* with plasmids. J. Mol. Biol..

[78] Webb J.T., Brown G.W. (1976). Some properties and occurrence of glutamine synthetase in fish. Comp. Biochem. Physiol. B.

[79] Gawronski J.D., Benson D.R. (2004). Microtiter assay for glutamine synthetase biosynthetic activity using inorganic phosphate detection. Anal Biochem..

[80] Smith P.K., Krohn R.I., Hermanson G.T., Mallia A.K., Gartner F.H., Provenzano M.D. (1985). Measurement of protein using bicinchoninic acid. Anal Biochem..

[81] Bartesaghi S., Wenzel J., Trujillo M., López M., Joseph J., Kalyanaraman B. (2010). Lipid peroxyl radicals mediate tyrosine dimerization and nitration in membranes. Chem. Res. Toxicol..

[82] Ellman G.L. (1959). Tissue sulfhydryl groups. Arch. Biochem. Biophys..

[83] Riordan J.F., Sokoloversusky M., Vallee B.L. (1967). The functional tyrosyl residues of carboxypeptidase A. Nitration with tetranitromethane. Biochemistry.

[84] Hawkins C.L., Morgan P.E., Davies M.J. (2009). Quantification of protein modification by oxidants. Free Radic. Biol. Med..

[85] Degendorfer G., Chuang C.Y., Mariotti M., Hammer A., Hoefler G., Hagglund P. (2018). Exposure of tropoelastin to peroxynitrous acid gives high yields of nitrated tyrosine residues, di-tyrosine cross-links and altered protein structure and function. Free Radic. Biol. Med..

[86] Gundry R.L., White M.Y., Murray C.I., Kane L.A., Fu Q., Stanley B.A. (2009). Preparation of proteins and peptides for mass spectrometry analysis in a bottom-up proteomics workflow. Curr. Protoc. Mol. Biol..

[87] Rappsilber J., Mann M., Ishihama Y. (2007). Protocol for micro-purification, enrichment, pre-fractionation and storage of peptides for proteomics using StageTips. Nat. Protoc..

[88] Rappsilber J., Ishihama Y., Mann M. (2003). Stop and go extraction tips for matrix-assisted laser desorption/ionization, nanoelectrospray, and LC/MS sample pretreatment in proteomics. Anal Chem..

[89] Cox J., Mann M. (2008). MaxQuant enables high peptide identification rates, individualized p.p.b.-range mass accuracies and proteome-wide protein quantification. Nat. Biotechnol..

[90] Samson A.L., Knaupp A.S., Kass I., Kleifeld O., Marijanovic E.M., Hughes V.A. (2014). Oxidation of an exposed methionine instigates the aggregation of glyceraldehyde-3-phosphate dehydrogenase. J. Biol. Chem..

[91] Eftink M.R. (1994). The use of fluorescence methods to monitor unfolding transitions in proteins. Biophys. J..

[92] Perez-Riverol Y., Bai J., Bandla C., Garcia-Seisdedos D., Hewapathirana S., Kamatchinathan S. (2022). The PRIDE database resources in 2022: a hub for mass spectrometry-based proteomics evidences. Nucleic Acids Res..

