# Supplementary material for: Multiple oxidative post-translational modifications of human glutamine synthetase mediate peroxynitrite-dependent enzyme inactivation and aggregation
Source: J Biol Chem. 2023 Jan 23;299(3):102941. doi: 10.1016/j.jbc.2023.102941 (PMC10011836; doi:10.1016/j.jbc.2023.102941)
Supplement: Supplemental Figures S1–S12 [file mmc1.docx]

**SUPPORTING INFORMATION**

**Multiple oxidative post-translational modifications of human glutamine synthetase mediate peroxynitrite-dependent enzyme inactivation and aggregation**

Nicolás Campolo^1^, Mauricio Mastrogiovanni^1^, Michele Mariotti^2^, Federico M. Issoglio^3,4^, Darío Estrin^5,6^, Per Hägglund^2^, Tilman Grune^7,8,9^, Michael J. Davies^2^, Silvina Bartesaghi^1^ and Rafael Radi^1,^**^[[1]](#footnote-1)^***

From the ^1^Departamento de Bioquímica and Centro de Investigaciones Biomédicas (CEINBIO), Facultad de Medicina, Universidad de la República, Uruguay; ^2^Department of Biomedical Sciences, Panum Institute, University of Copenhagen, Copenhagen, Denmark; ^3^CONICET-Universidad de Buenos Aires, Instituto de Química Biológica de la Facultad de Ciencias Exactas y Naturales (IQUIBICEN), Buenos Aires, Argentina; ^4^Instituto de Tecnologia Química e Biológica António Xavier, Universidade Nova de Lisboa (ITQB NOVA), Oeiras, Portugal; ^5^CONICET-Universidad de Buenos Aires, Instituto de Química Física de los Materiales, Medio Ambiente y Energía (INQUIMAE), Buenos Aires, Argentina; ^6^Universidad de Buenos Aires, Facultad de Ciencias Exactas y Naturales, Departamento de Química Inorgánica, Analítica y Química Física, Buenos Aires, Argentina; ^7^Department of Molecular Toxicology, German Institute of Human Nutrition, Potsdam-Rehbrücke, 14558, Nuthetal, Germany; ^8^German Center for Cardiovascular Research (DZHK), 10117, Berlin, Germany; ^9^Department of Physiological Chemistry, Faculty of Chemistry, University of Vienna, 1090 Vienna, Austria.


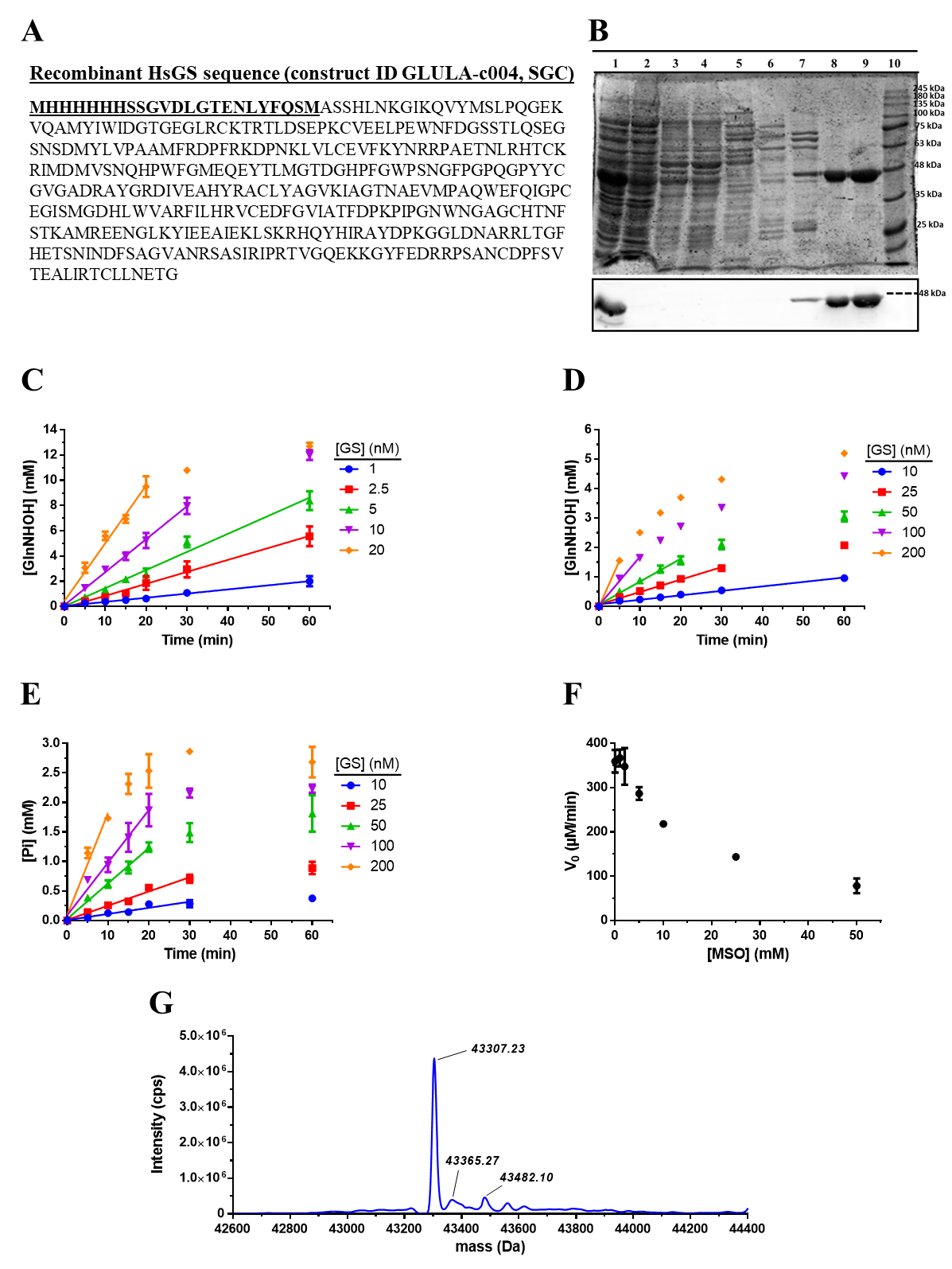


**Figure S1. Characterization of purified recombinant HsGS. (A)** Amino acid sequence of the HsGS construct used (N-terminal tag is shown in bold and underlined). **(B)** Reducing SDS-PAGE (above) and anti-GS western-blot (below) analysis of the purification process of recombinant HsGS (above). 1: bacterial lysate; 2: flow-through; 3-4: 10 mM imidazole washes; 5: 25 mM imidazole wash; 6: 50 mM imidazole wash; 7: 100 mM imidazole wash; 8-9: 500 mM imidazole eluate (5 and 10 µg); 10: molecular weight marker. **(C-E)** Time-course of product formation for different HsGS concentrations through: **(C)** the γ-glutamyl transferase assay; **(D)** the ATP-dependent synthesis of γ-glutamyl hydroxamate from glutamate and NH_2_OH; **(E)** the physiological glutamine synthesis. **(F)** Inhibition of HsGS by the specific inhibitor L-methionine sulfoximine (MSO) measured by the the γ-glutamyl transferase assay. **(G)** Full-protein MS characterization for molecular weight determination. Data are shown as mean ± sd (n = 3).


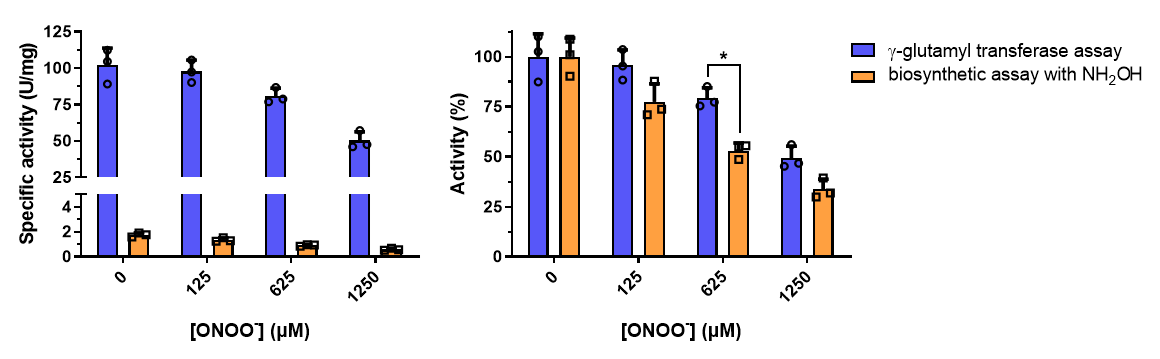


**Figure S2. Comparison of HsGS inactivation by ONOO^-^ through two different activity assays.** HsGS (0.5 mg mL^-1^) was exposed to 125-1250 µM ONOO^-^ in phosphate buffer pH 7.3 and then aliquots were taken for activity measurements. For the γ-glutamyl transferase assay, 10 nM HsGS decamer was incubated with substrate mix for 15 min at 37ºC; for the biosynthetic assay with NH_2_OH, 50 nM HsGS decamer was used instead, and incubated at 37ºC for 30 min with the corresponding substrate mix. **(A)** Specific HsGS activity measured through each assay. **(B)** Relative activity expressed as % of the control activity (0 µM ONOO^-^) for each assay. Data are shown as mean ± sd (n = 3). ***,** p ˂ 0.05 by unpaired t test analysis.

**Figure S3.** **Densitometric quantitation of the reducing SDS-PAGE analysis of HsGS exposed to ONOO^-^.** The intensity of each of the different species that appeared in the reducing SDS-PAGE analysis (Fig. 1B) was measured by densitometry from the scanned gel images using the Image Studio Software (LiCor). Intensity values were normalized to the intensity of the monomer band of the control (0 µM ONOO^-^) sample and expressed as % of it. Relative intensities of the monomer bands are plotted on the left Y-axis; relative intensities of the dimer (D), trimer (T) and higher molecular weight species (HMW) bands are plotted on the right Y-axis. Data are shown as mean ± sd (n = 3).


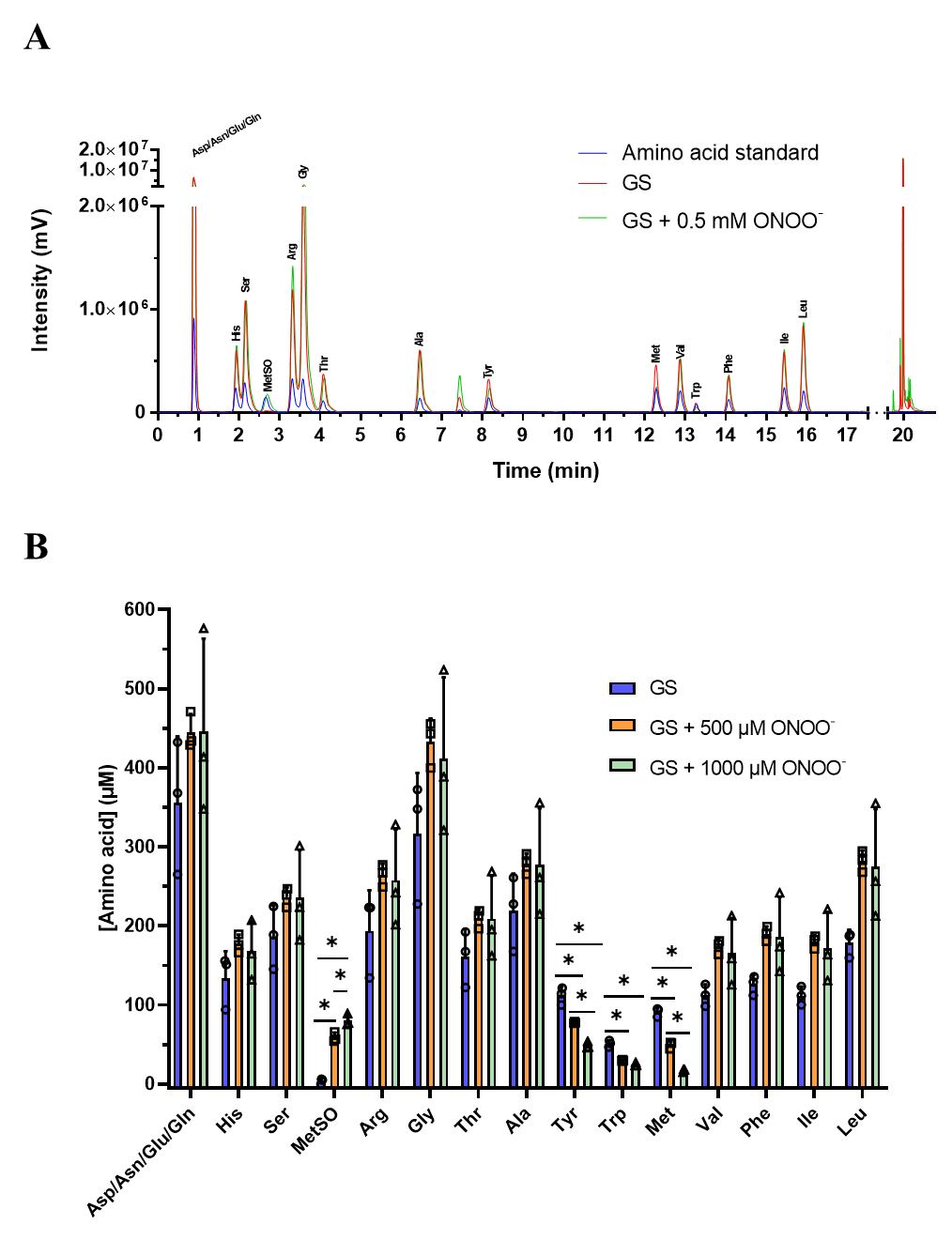


**Figure S4. Amino acid analysis of HsGS hydrolysates by UHPLC. (A)** Representative chromatograms of control and ONOO^-^-treated HsGS, along with amino acid standard mix. **(B)** Amino acid concentrations obtained for all the measured species. Data are shown as mean ± sd (n = 3). *, p ˂ 0.05 by one-way ANOVA with Tukey post hoc analysis.

**Figure S5. MS1-filtering estimation of the relative modification levels of specific residues.** MS1-filtering analysis was performed in the Skyline software from the raw data obtained from the nLC-MS/MS characterization of treated HsGS samples and the peptide identification list obtained for each sample through the MaxQuant software. The area of the peaks corresponding to the native and modified peptides were obtained and used for estimating the percentage of modification of each residue. Data are shown as mean ± sd (n = 3).


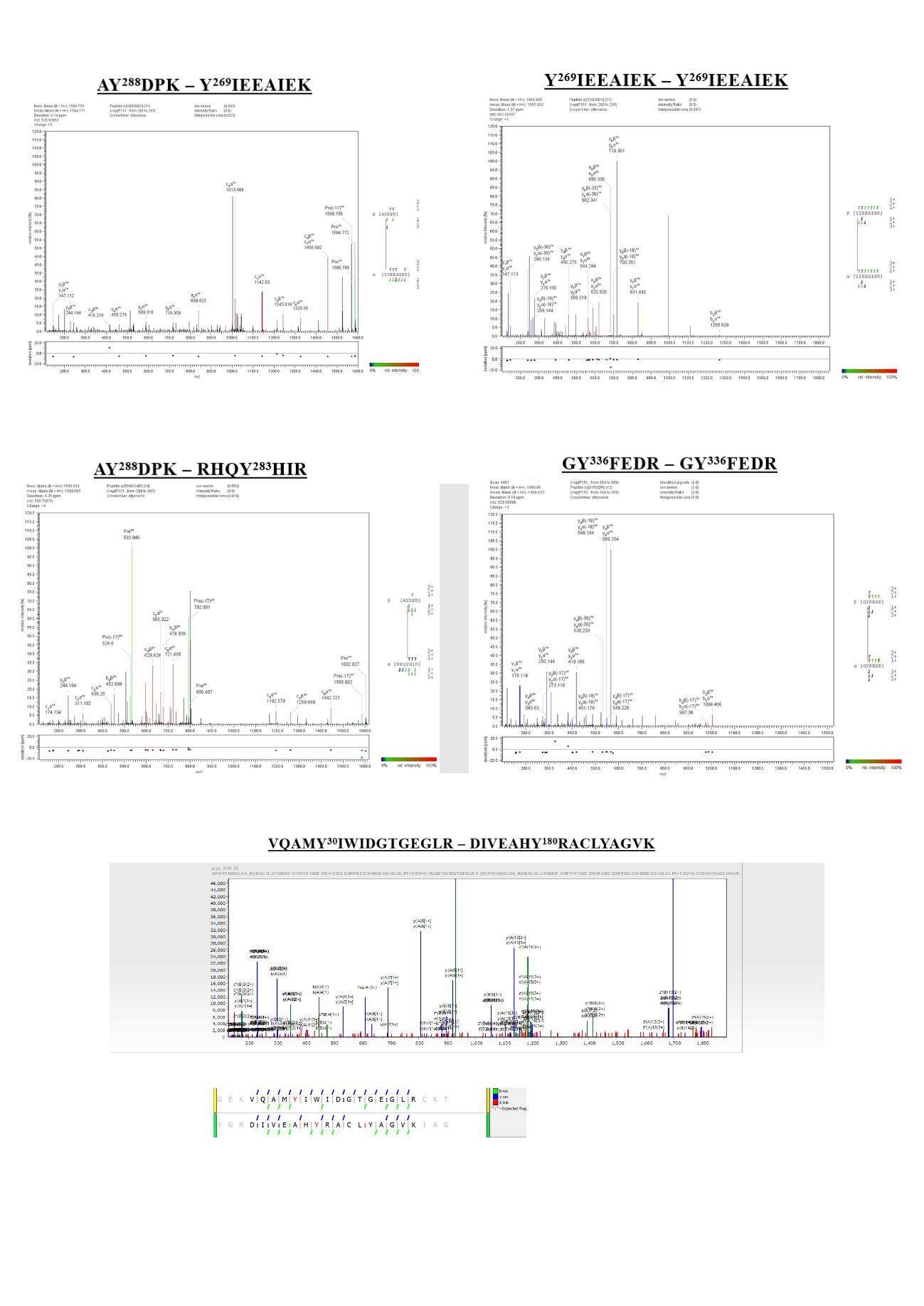


**Figure S6. Identification of DiTyr HsGS cross-links.** MS/MS spectra of the five identified DiTyr protein cross-links through the H_2_^18^O nLC-MS/MS analysis of trypsin-digested peroxynitrite-treated HsGS.


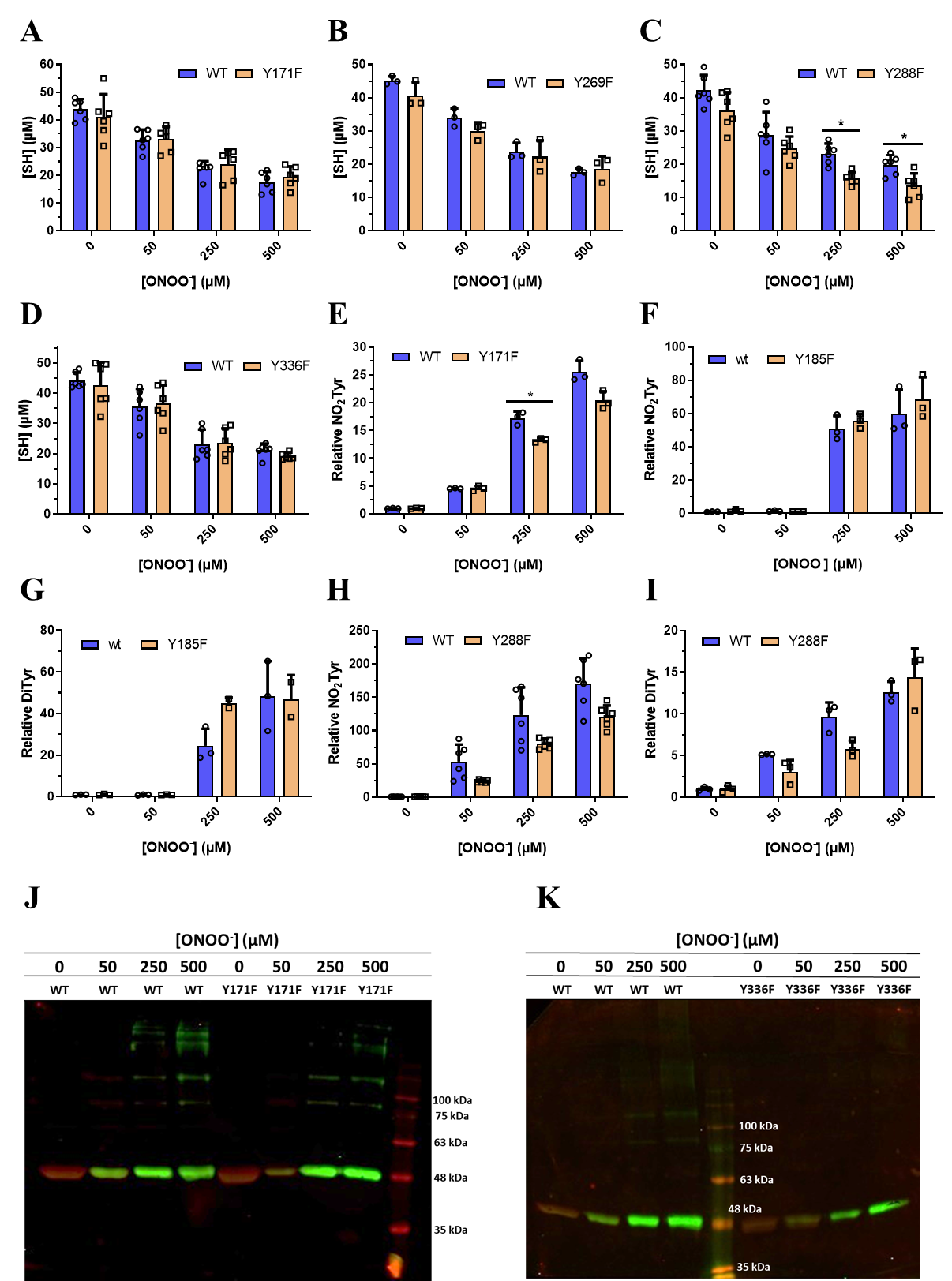


**Figure S7. Comparative biochemical characterization of ONOO^-^-treated HsGS Tyr→Phe mutants. (A-D)** Thiol quantification through reaction with DTNB. **(E-I)** Relative NO_2_Tyr and DiTyr levels assessed by dot-blot for some of the mutants. **(J-K)** Western-blot analysis of tyrosine nitration of the Y171F and Y336F mutants exposed to ONOO^-^ (green: anti-NO_2_Tyr; red: anti-GS). Data are shown as mean ± sd (n = 3-6). *, p ˂ 0.05 by unpaired t test analysis.


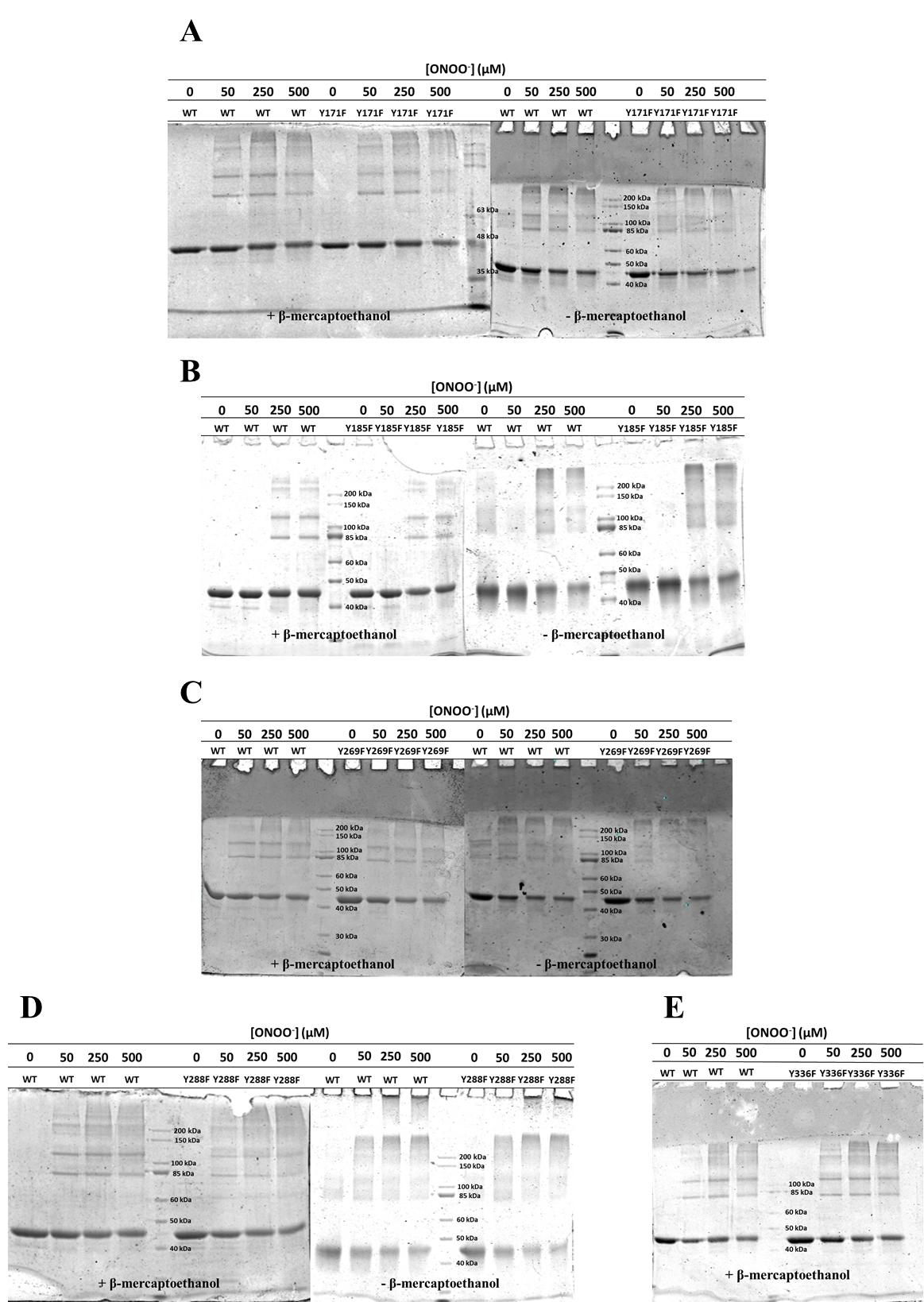


**Figure S8. Comparative SDS-PAGE analysis of ONOO^-^-treated HsGS Tyr→Phe mutants.** After exposing wt and mutants of HsGS to ONOO^-^, samples were comparatively analyzed by reducing or non-reducing SDS-PAGE. **(A)** Y171F variant; **(B)** Y185F variant; **(C)** Y269F variant; **(D)** Y288F variant; **(E)**: Y336F variant.


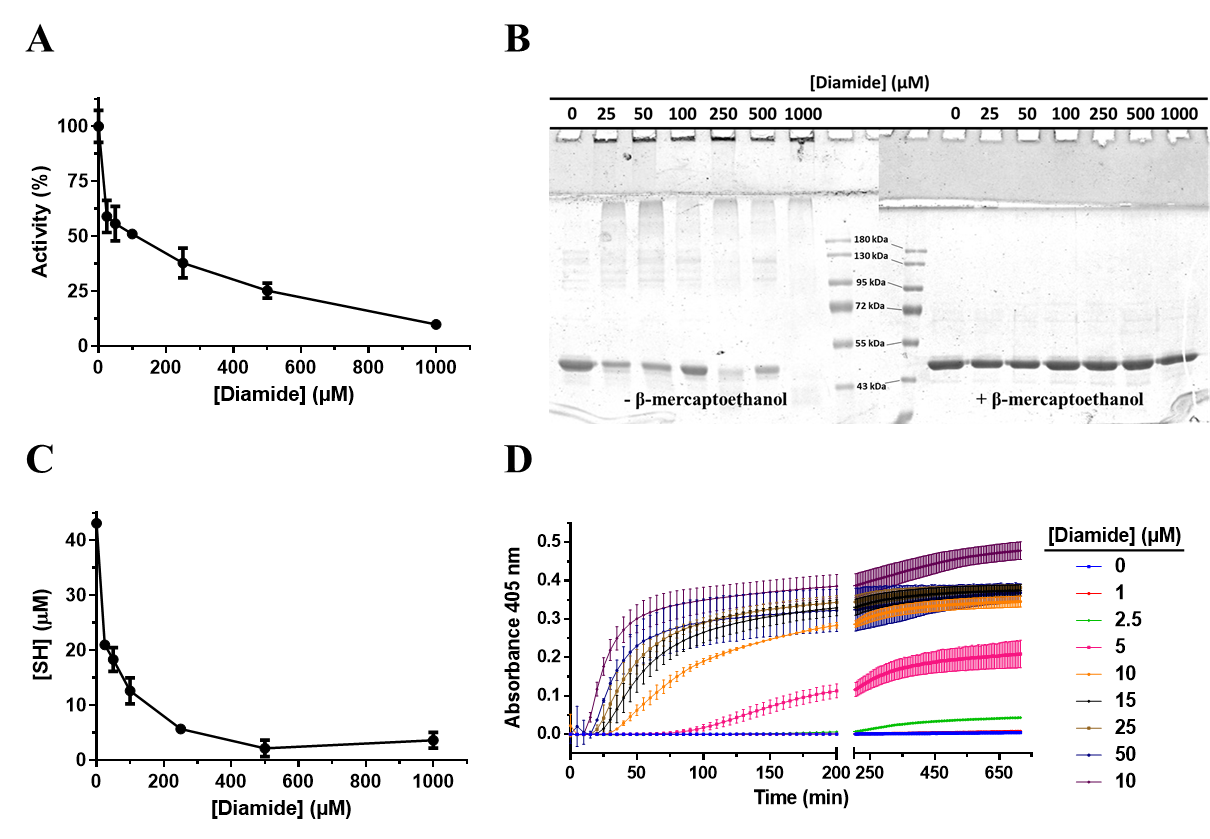


**Figure S9. Oxidation of HsGS by diamide.** HsGS (0.2 mg mL^-1^) was incubated with diamide (25-1000 µM) at 37ºC for 1 h in phosphate buffer pH 7.3. After incubation, aliquots were taken for: **(A)** activity assays (100 % = 72.6 U/mg), **(B)** reducing and non-reducing SDS-PAGE analysis and **(C)** thiol quantification. **(D)** Diamide-induced HsGS aggregation was assessed through turbidity measurements at 405 nm for 12 h at 37ºC in a 96-well plate reader. Data are shown as mean ± sd (n = 3).


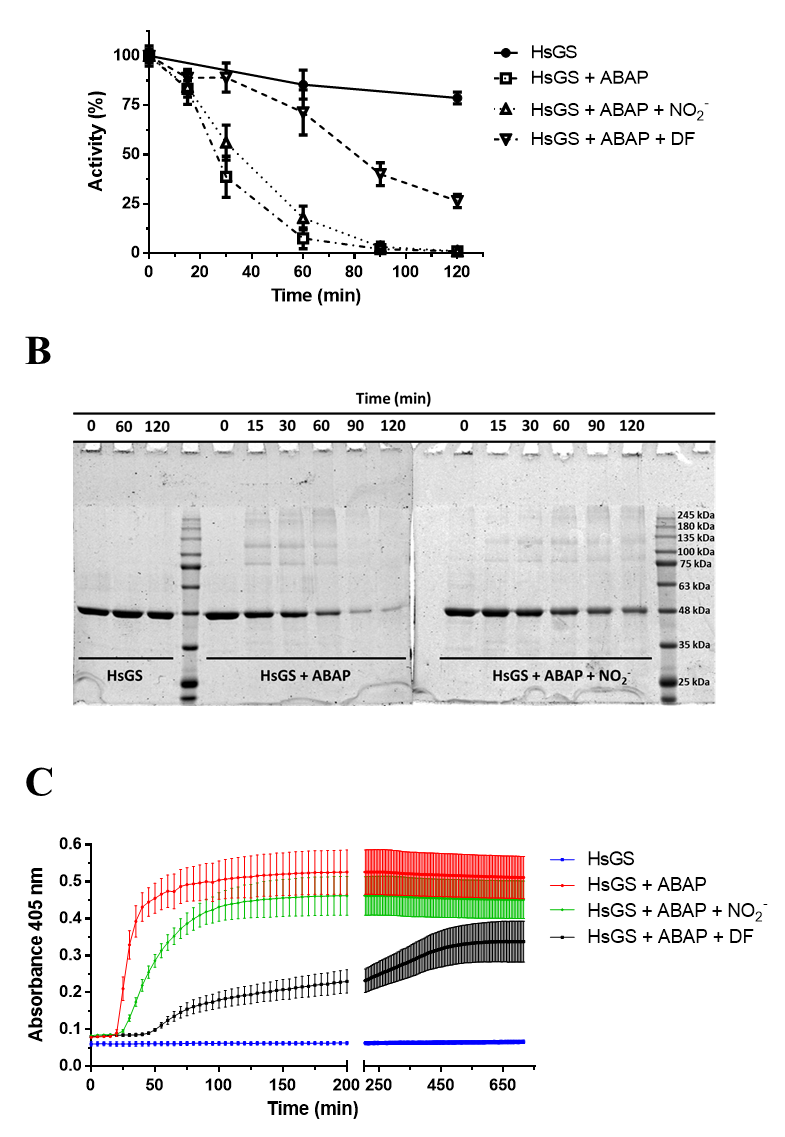


**Figure S10. ABAP-induced HsGS oxidation.** HsGS (0.2 mg mL^-1^) was incubated with 10 mM ABAP at 37ºC in KPi buffer pH 7.3 containing 0.1 M KCl and 0.1 mM DTPA for 2 h. Aliquots were taken at different times and analyzed through: **(A)** activity measurements (100 % = 66.2 U/mg) and **(B)** reducing SDS-PAGE. **(C)** ABAP-induced aggregation was followed through turbidity measurements after incubation of the enzyme with 10 mM ABAP at 37ºC for 12 h. Data are shown as mean ± sd (n = 3).


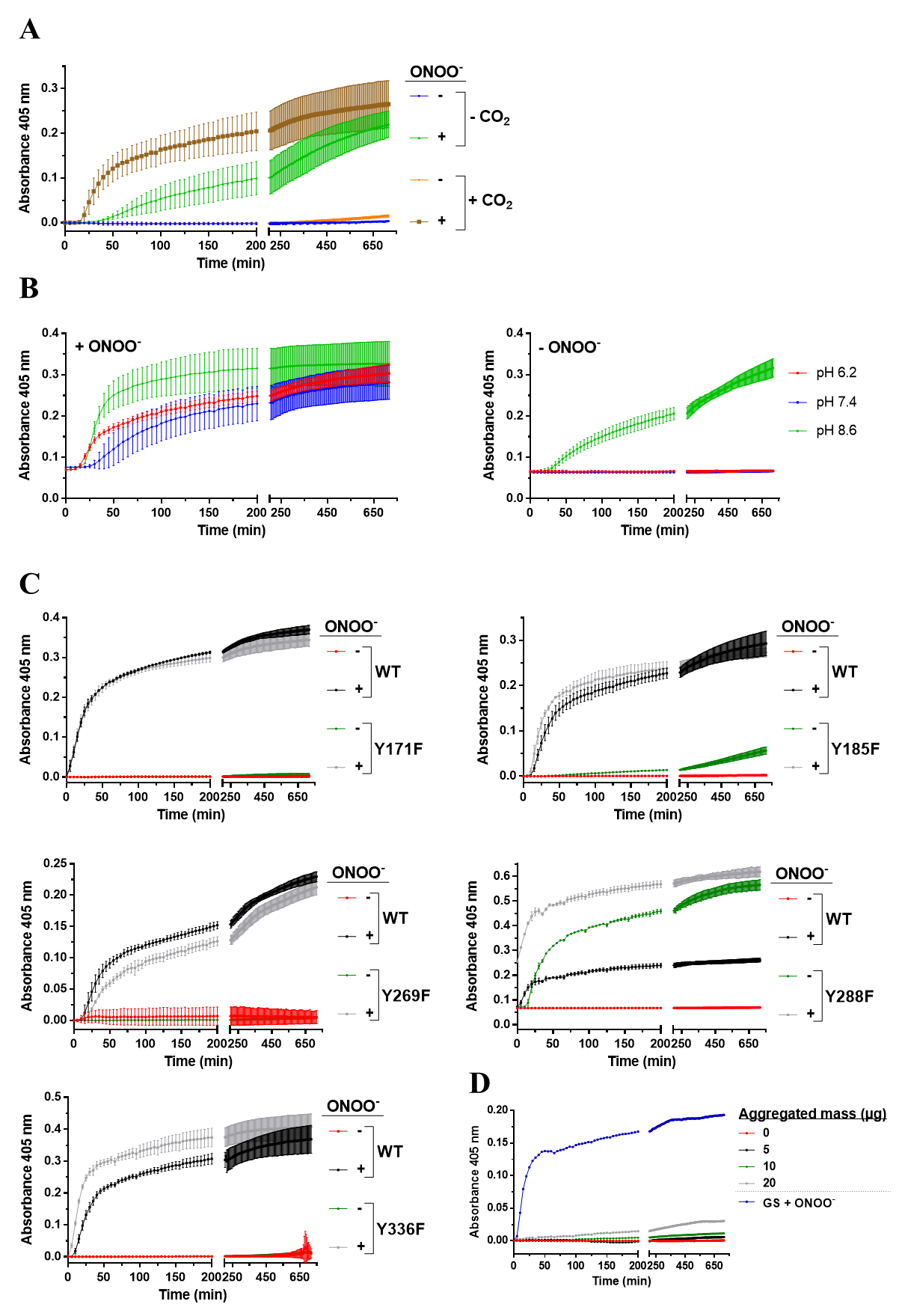


**Figure S11. Aggregation of HsGS by ONOO^-^ under different conditions and analysis of the Tyr→Phe mutants. (A)** Comparative HsGS aggregation by 250 µM ONOO^-^ in the absence or presence of CO_2_. **(B)** ONOO^-^-induced HsGS aggregation at different pH values (exposure to 500 µM ONOO^-^), as well as the corresponding controls without ONOO^-^ addition. **(C)** Aggregation of the five HsGS mutants (Y171F, Y185F, Y269F, Y288F and Y336F) in comparison to the WT enzyme after their exposure to a single 500 µM ONOO^-^ bolus. **(D)** HsGS (0.2 mg mL^-1^) was incubated with different amounts of pre-aggregated HsGS (0-20 µg) and incubated at 37ºC for 12 h; aggregation was followed through turbidity measurements. Data are shown as mean ± sd (n = 3).


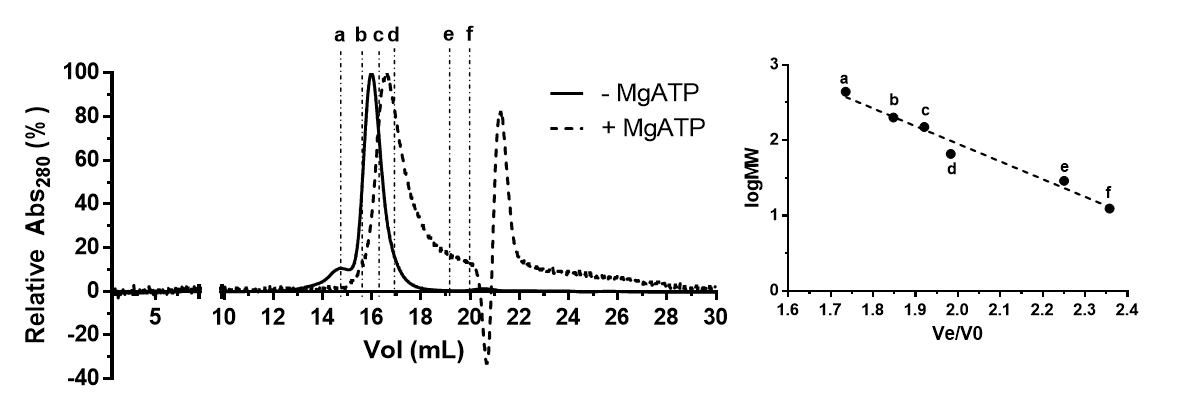


**Figure S12. Effect of MgATP binding on the oligomerization state of HsGS by gel filtration.** The HsGS (4.0 mg mL^-1^) oligomerization state was studied on a Superdex 200 10/300 GL gel filtration column either without (- MgATP) or with (+ MgATP) 5 mM ATP and 10 mM MgCl_2_. The elution volumes (V_e_) of the molecular weight markers are indicated with vertical lines (**left**). Blue dextran was used for obtaining the void volume (V_0_). The linear regression of the plot logMW vs Ve/V0 allowed for the estimation of MW (**right**). The proteins used for MW calibration were: **a**, ferritin (440 kDa); **b**, β-amylase (200 kDa); **c**, alcohol dehydrogenase (150 kDa); **d**, bovine serum albumin (66 kDa); **e**, carbonic anhydrase (29 kDa); **f**, cytochrome c (12 kDa).

1. * For correspondence: Rafael Radi, rradi@fmed.edu.uy [↑](#footnote-ref-1)
